# Analysis of two birth tissues provides new insights into the epigenetic landscape of neonates born preterm

**DOI:** 10.1186/s13148-018-0599-4

**Published:** 2019-02-11

**Authors:** Yonghui Wu, Xinyi Lin, Ives Yubin Lim, Li Chen, Ai Ling Teh, Julia L. MacIsaac, Kok Hian Tan, Michael S. Kobor, Yap Seng Chong, Peter D. Gluckman, Neerja Karnani

**Affiliations:** 1 Singapore Institute for Clinical Sciences, A*STAR, 30 Medical Drive, Singapore, 117609 Singapore; 2 Duke-NUS Medical School, Singapore, Singapore; 3 Department of Medical Genetics, Centre for Molecular Medicine and Therapeutics, Child and Family Research Institute, University of British Columbia, Vancouver, Canada; 4 KK Women’s and Children’s Hospital, Singapore, Singapore; 5 Department of Obstetrics and Gynaecology, Yong Loo Lin School of Medicine, National University of Singapore, Singapore, Singapore; 6 Centre for Human Evolution, Adaptation and Disease, Liggins Institute, University of Auckland, Auckland, New Zealand; 7 Department of Biochemistry, Yong Loo Lin School of Medicine, National University of Singapore, Singapore, Singapore

**Keywords:** Epigenome wide association study, Preterm birth, Gestational age, Tissue specificity, DNA methylation, Neonate

## Abstract

**Background:**

Preterm birth (PTB), defined as child birth before completion of 37 weeks of gestation, is a major challenge in perinatal health care and can bear long-term medical and financial burden. Over a million children die each year due to PTB complications, and those who survive can face developmental delays. Unfortunately, our understanding of the molecular pathways associated with PTB remains limited. There is a growing body of evidence suggesting the role of DNA methylation (DNAm) in mediating the effects of PTB on future health outcomes. Thus, epigenome-wide association studies (EWAS), where DNAm sites are examined for associations with PTB, can help shed light on the biological mechanisms linking the two.

**Results:**

In an Asian cohort of 1019 infants (68 preterm, 951 full term), we examined and compared the associations between PTB and genome-wide DNAm profiles using both cord tissue (*n* = 1019) and cord blood (*n* = 332) samples on Infinium HumanMethylation450 arrays. PTB was significantly associated (*P* < 5.8e−7) with DNAm at 296 CpGs (209 genes) in the cord blood. Over 95% of these CpGs were replicated in other PTB/gestational age EWAS conducted in (cord) blood. This replication was apparent even across populations of different ethnic origin (Asians, Caucasians, and African Americans). More than a third of these 296 CpGs were replicated in at least 4 independent studies, thereby identifying a robust set of PTB-linked epigenetic signatures in cord blood. Interrogation of cord tissue in addition to cord blood provided novel insights into the epigenetic status of the neonates born preterm. Overall, 994 CpGs (608 genes, *P* < 3.7e−7) associated with PTB in cord tissue, of which only 10 of these CpGs were identified in the analysis using cord blood. Genes from cord tissue showed enrichment of molecular pathways related to fetal growth and development, while those from cord blood showed enrichment of immune response pathways. A substantial number of PTB-associated CpGs from both the birth tissues were also associated with gestational age.

**Conclusions:**

Our findings provide insights into the epigenetic landscape of neonates born preterm, and that its status is captured more comprehensively by interrogation of more than one neonatal tissue in tandem. Both these neonatal tissues are clinically relevant in their unique ways and require careful consideration in identification of biomarkers related to PTB and gestational age.

**Trial registration:**

This birth cohort is a prospective observational study designed to study the developmental origins of health and disease, and was retrospectively registered on 1 July 2010 under the identifier NCT01174875.

**Electronic supplementary material:**

The online version of this article (10.1186/s13148-018-0599-4) contains supplementary material, which is available to authorized users.

## Background

Preterm birth (PTB), defined as delivery of the offspring before completion of 37 weeks of gestation, is a major public health problem that exerts a significant disease burden globally [1]. In 2016, World Health Organization estimated 15 million babies (at least 1 in 10 babies) to be born preterm annually, and that these numbers are rising each year [2]. PTB is associated with developmental delays, and infants born preterm are at an increased risk of mortality from infancy to adulthood due to the onset of various chronic health problems [3, 4]. However, the biological pathways underlying the associations between PTB and future health remain elusive [5, 6]. Epigenetic mechanisms play a critical role in regulating cell lineage commitment and fetal programing and are highly sensitive to in utero perturbations. Any interference with the epigenetic settings within the cell or its developmental state can have life-long impact on the health of the offspring. Thus, epigenome-wide association studies (EWAS) related to PTB [7–12] can help elucidate the biological mechanisms linking the two [13].

There is a growing body of evidence suggesting the influence of PTB on neonatal epigenome through DNA methylation (DNAm) [7, 8, 13–18]. Earlier efforts in interrogating DNAm changes in association with PTB typically focused on candidate regions of the epigenome [19, 20] or were conducted in smaller sample sizes [7–10, 18]. Recently, some research groups have conducted EWAS of gestational age (GA), with some using larger sample sizes. Schroeder et al. [21] reported and replicated the association between DNAm and GA at CpG sites in 25 genes, genes previously implicated in labor and delivery and adverse health outcomes. Lee et al. [22] reported DNAm at three regions associated with GA, regions located near genes that play key roles in fetal development (*NFIX*, *RAPGEF2*, *MSRB3*). Bohlin et al. [11] and Simpkin et al. [12] reported DNAm at 5474 CpG sites and 224 CpG sites to associate with GA, respectively. Though the total sample sizes in these GA EWAS were larger, with the exception of Bohlin et al. [11], the number of preterm infants in the analyses did not exceed 30.

While earlier studies have made significant progress in identifying DNAm perturbations associated with PTB/GA and enhanced our understanding of the epigenetic processes associated with PTB, a few important considerations remain. First, as earlier investigations were primarily conducted in Caucasian and/or African American populations, it is unclear how these findings hold in an Asian population. Second, earlier work primarily focused on examination of DNAm in infant cord blood [7, 9–12, 16, 21, 22], but there have been no studies done on cord tissue. Since cord tissue and cord blood originate from different cell lineages, each tissue potentially reveals unique perspectives within the preterm scenario. Pertinently, our earlier work has demonstrated that neonate EWAS conducted using infant cord tissue can give very distinct findings from those conducted in cord blood [23]. Hence, the two tissues together capture a better understanding of the epigenetic alterations induced by a suboptimal fetal environment. Here, we present the first EWAS of PTB conducted in an Asian cohort, where we examine and compare the associations of PTB with DNAm in both infant cord tissue and cord blood.

## Results

### Study population

This study involved 1019 infants from live singleton births, of which 68 infants were born preterm (Additional file 1: Figure S1A). Summary statistics of these infants are provided in Additional file 2: Table S1. The ethnic distribution of study subjects with available cord tissue samples was 58% Chinese, 25% Malay, and 17% Indian. Fifty-three percent of the infants were male. The difference in the distributions of ethnicity (*P* = 0.88) and sex (*P* = 0.90) of the infants in preterm vs. term groups was not statistically significant. We interrogated DNAm profiles derived from infant cord tissue and cord blood using the Infinium HumanMethylation450 array. DNAm data was available for all 1019 infants for cord tissue and in a subset of infants for cord blood (332 infants, including 31 preterm infants, Additional file 2: Table S2, Additional file 1: Figure S1B). Similarly, the distributions of infants with cord blood samples in preterm vs. term groups were not significantly different with respect to ethnicity (*P* = 0.47) and infant sex (*P* = 0.58). After quality control and elimination of CpGs with low variability, 134,676 and 85,624 CpGs were retained for subsequent analyses in cord tissue and cord blood, respectively.

### Cord tissue reflected extensive associations between PTB and infant DNAm

**Fig. 1:**
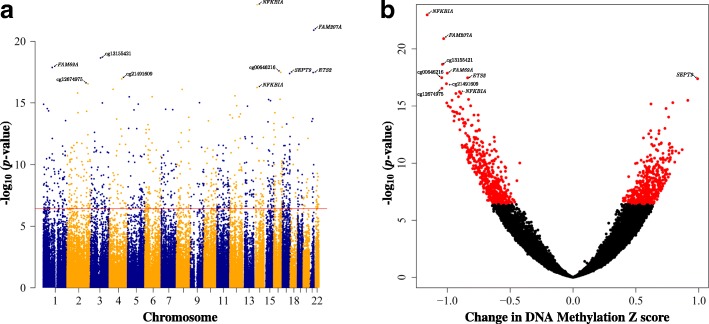
Preterm births (PTB) were associated with global alterations in infants’ cord tissue DNA methylation. **a** Manhattan plot and **b** volcano plot illustrating the relationship of the 134,676 infant cord tissue CpGs analyzed with respect to PTB. The top 10 CpGs with the smallest *P* values are indicated on both plots and labeled with the gene it is associated with or CpG identifier if the CpG lies within an intergenic region. Points on each plot represent individual CpGs which in **a** have genomic locations on the horizontal axis with alternating colors representing different chromosomes and in **b** have the change in DNA methylation *Z*-score on the horizontal axis. The red horizontal line in **a** represents the Bonferroni threshold (*P* < 3.7 × 10^−7^). Nine hundred ninety-four infant cord tissue CpGs were found to significantly associate with PTB and are indicated as red points in **b**. In both plots, the vertical axis represents the negative log10 *P* values with respect to PTB, adjusted for infant sex, ethnicity, cell-type proportions, bisulfite conversion batch, and DNA extraction batch

We examined the association between cord tissue DNAm and PTB and identified 994 CpGs to be significantly associated with PTB using a Bonferroni multiple testing correction (*P* < 3.7e−7; Fig. 1, Additional file 2: Table S3). The percentage of PTB-associated CpGs in hypomethylation (49%, 492 CpGs) and hypermethylation (51%, 502 CpGs) groups was almost equal (Fig. 1b), and their absolute effect size estimates (change in cord tissue DNAm *Z*-score with respect to PTB status) ranged from 0.40 to 1.16 (Additional file 2: Table S3). These 994 CpGs mapped to 608 unique genes, with the top most statistically significant CpGs mapping to several transcription factors such as nuclear factor of kappa light polypeptide gene enhancer in B cells inhibitor, alpha (*NFKBIA*); ETS proto-oncogene 2, transcription factor (*ETS2*); and potential cell cycle control factors such as Septin 9 (*SEPT9*), family with sequence similarity 69 member A (*FAM69A*), and sequence similarity 207 member A (*FAM207A*). These 994 cord tissue CpGs remained largely statistically significant in sensitivity analyses (Additional file 1: Figure S2, Additional file 2: Table S3), with 79–87% remaining statistically significant after Bonferroni adjustment and 99–100% reflecting nominal significance with *P* value < 10^−4^.

### Cord blood reflected extensive associations between PTB and infant DNAm

**Fig. 2:**
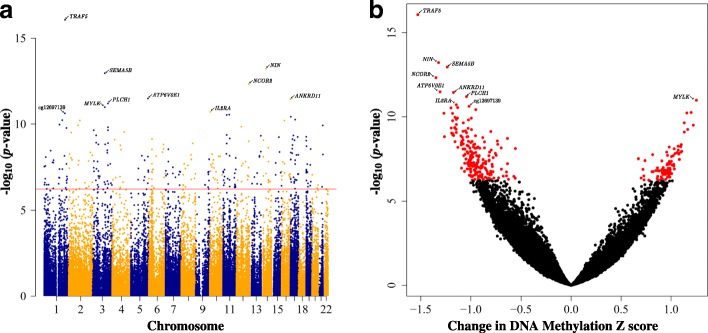
Preterm births (PTB) were associated with global alterations in infants’ cord blood DNA methylation. **a** Manhattan plot and **b** volcano plot illustrating the relationship of the 85,624 infant cord blood CpGs analyzed with respect to PTB. The top 10 CpGs with the smallest *P* values are indicated on both plots and labeled with the gene it is associated with or CpG identifier if the CpG lies within an intergenic region. Points on each plot represent individual CpGs which in **a** have genomic locations on the horizontal axis with alternating colors representing different chromosomes and in **b** have the change in DNA methylation *Z*-score on the horizontal axis. The red horizontal line in **a** represents the Bonferroni threshold (*P* < 5.8 × 10^−7^). Two hundred ninety-six infant cord blood CpGs were found to significantly associate with PTB and are indicated as red points in **b**. In both plots, the vertical axis represents the negative log10 *P* values with respect to preterm birth status, adjusted for infant sex, ethnicity, cell-type proportions, and bisulfite conversion batch

We further interrogated the association between cord blood DNAm and PTB in 332 infants (these 332 infants are a subset of the 1019 infants). After adjusting for multiple testing using a Bonferroni correction (*P* < 5.8e−7; Fig. 2, Additional file 2: Table S4), 296 CpGs in 209 unique genes were identified to significantly associate with PTB in infant cord blood. These CpGs had absolute effect size estimates (change in cord blood DNAm *Z*-score with respect to PTB status) ranging from 0.55 to 1.53 (Additional file 2: Table S4). Ten CpGs overlapped between the cord blood (296 CpGs) and cord tissue (994 CpGs) analyses. The top most statistically significant CpGs on this list included immune response and signaling genes such as TNF receptor-associated factor 5 (*TRAF5*), nuclear receptor corepressor 2 (*NCOR2*), myosin light-chain kinase (*MYLK*), and interleukin 2 receptor subunit alpha (*IL2RA*) and phospholipase C eta 1 (*PLCH1*). These 296 cord blood CpGs remained largely statistically significant in sensitivity analyses (Additional file 1: Figure S3, Additional file 2: Table S4), with 94–97% remaining Bonferroni significant and 100% reflecting nominal significance with *P* value < 10^−4^. In contrast to our observation in cord tissue, relatively lower number of CpGs (31%) showed hypomethylation in response to preterm in cord blood (Fig. 2b).

### Majority of PTB-associated CpGs in cord blood are replicated in other PTB/GA EWAS

**Fig. 3:**
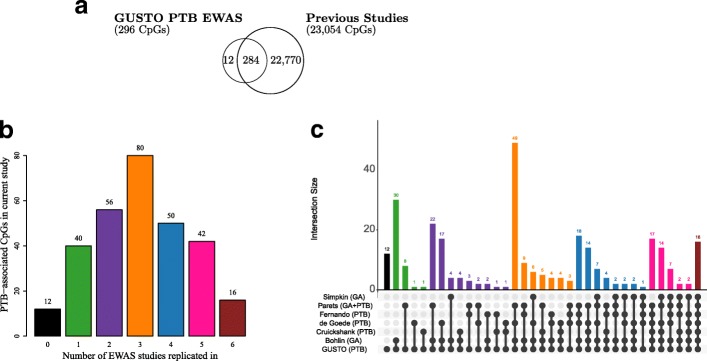
Cord blood CpGs previously reported in association with gestational age (GA) or preterm births (PTB). **a** The Venn diagram shows the relationship between cord blood CpGs previously reported to be significantly associated with gestational age or PTB in relation to PTB-associated CpGs in the current study. **b** The bar graph shows the reproducibility of the 296 PTB-associated cord blood CpGs in the current study. The vertical axis gives the number of PTB-associated cord blood CpGs in the current study, while the horizontal axis gives the number of earlier GA/PTB epigenome-wide association studies (EWAS) our PTB-associated cord blood CpGs are replicated in. Bar graph colors are representative of the number of earlier studies our PTB-associated CpGs replicated in black (0), green (1), purple (2), orange (3), blue (4), pink (5), and brown (6). **c** This UpSet plot further breaks down the replication of our PTB-associated CpGs in the earlier studies. Each column represents the number of CpGs, for each unique intersection of the current study (GUSTO) with other studies, as indicated by the gray dot and connecting line. Intersection sets with no CpGs are not shown on the plot

Since our study was conducted in an Asian population, we compared our cord blood EWAS findings with six previously reported studies in Caucasian and African American populations using the same Infinium HumanMethylation450 platform [7–12]. We consider a CpG to be replicated if it was reported in at least one of the previously conducted studies using the same Infinium HumanMethylation450 platform [7–12]. Of the 296 CpGs identified in our study, > 95% (284 CpGs) could be replicated in at least 1 of the previous studies and > 80% (244 CpGs) in at least 2 of the previous studies (Fig. 3, Additional file 2: Table S5 and S6), indicating robustness of the findings and a commonality in PTB associations across various ethnicities. Sixteen of these CpGs (12 genes) were reproducible in all 6 earlier independent studies, identifying robust epigenetic signatures of PTB. A subset of CpGs (22,770) from the previous 6 studies (Fig. 3) [7–12] were not identified in our study; however, these CpGs in general showed low reproducibility as only 15% of these were replicated in at least 1 of the studies and the remainder 85% showed no replication (Additional file 1: Figure S4). Nevertheless, the replicated CpGs from the earlier studies that are not identified in our study are worthy of further consideration. We also applied the DNAm GA clocks published by Knight et al. [24] and Bohlin et al. [11] to predict GA in our study samples (Additional file 1: Figure S5). For both epigenetic clocks, the performance in cord blood (correlation = 0.52 for Knight et al. clock and correlation = 0.72 for Bohlin et al. clock, *n* = 301 term samples only) was better than the performance in cord tissue (correlation = 0.13 for Knight et al. clock and correlation = 0.16 for Bohlin et al. clock, *n* = 951 term samples only). The better performance in cord blood vs. cord tissue was not unexpected as both epigenetic clocks were derived using (cord) blood samples. Similar to an earlier report [25], the performance improved when preterm infants were included, with better performance for cord blood (correlation = 0.69 for Knight et al. clock and correlation = 0.85 for Bohlin et al. clock, *n* = 332) than cord tissue (correlation = 0.15 for Knight et al. clock and correlation = 0.22 for Bohlin et al. clock, *n* = 1019).

### DNA methylomes of cord blood and cord tissue respond differently to PTB

For CpGs significantly associated with PTB in at least one tissue, we also assessed whether there was evidence of tissue-dependent effects. For the 994 PTB-associated CpGs from cord tissue, 546 CpGs were removed from the cord blood dataset due to quality control filtering (426 of them were due to low inter-individual variation); for the remainder 448 CpGs, majority of the CpGs (143 at *P* < 1e−4, 310 at *P* < 0.05) showed evidence of tissue-dependent effects (Additional file 2: Table S7). Similarly for the 296 PTB-associated CpGs from cord blood, 102 CpGs were removed from the cord blood dataset due to quality control filtering (29 of them were due to low inter-individual variation); for the remainder 194 CpGs, majority of the CpGs (126 at *P* < 1e−4, 184 at *P* < 0.05) showed evidence of tissue-dependent effects (Additional file 2: Table S8).

### DNAm status of genes affected by PTB in the two neonatal tissues represents distinct biological processes

**Fig. 4:**
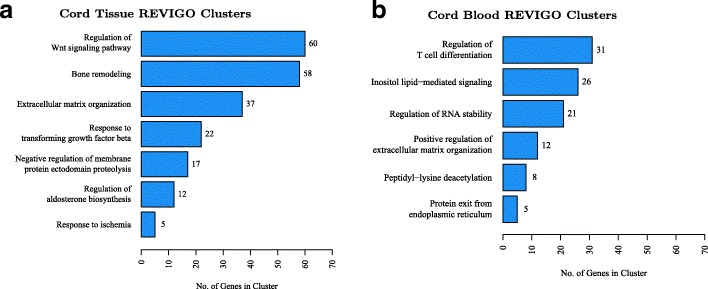
**a**, **b** REVIGO summarized Gene Ontology Clusters with respect to preterm birth (PTB)-associated CpGs in both cord tissue and cord blood. Gene ontology (GO) enrichment was performed on PTB-associated CpGs in both cord tissue and cord blood for each tissue separately using missMethyl. REVIGO was then used to reclassify the biological process-related enriched GO terms (parent GO term containing under 300 genes, semantic similarity measure between each GO term < 0.7). Cord tissue CpGs had 10 GO clusters from 41 unique GO terms, while cord blood CpGs had 10 GO clusters from 43 unique GO terms. GO clusters with 5 or more genes are represented by the bar graphs, with plots on the left and right corresponding to cord tissue and cord blood respectively. The vertical axis of the bar graphs represents the REVIGO cluster names, while the horizontal axis represents the number of genes in the REVIGO cluster containing at least one significantly associated CpG

We performed gene ontology analyses on the 994 cord tissue CpGs and 296 cord blood CpGs found to be significantly associated with PTB. Top gene ontology terms enriched with respect to cord tissue reflected biological processes primarily involved in fetal growth and development, i.e., Wnt signaling, bone remodeling, and extracellular matrix organization (Fig. 4, Additional file 1: Figure S6 and S7, Additional file 2: Table S9). In contrast, cord blood reflected regulation of T cell differentiation, inositol lipid-mediated signaling, and regulation of RNA stability (Fig. 4, Additional file 1: Figure S8 and S9, Additional file 2: Table S10). These results are consistent with the fact that variable CpGs in each tissue tend to over-represent certain pathways. This also is clearly evident from the gene ontology analysis of all variable CpGs identified on the Infinium HumanMethylation450 platform from each tissue (Additional file 1: Figure S10 and S11).

### Majority of PTB-associated CpGs in cord tissue and cord blood were also associated with GA

Lastly, we also examined the associations between GA and DNAm in each tissue. In this analysis, GA was modeled as a continuous variable instead of a binary variable (preterm vs. term). After adjustment for multiple testing, 4075 CpGs (*P* < 3.7e−7) were significantly associated with GA in cord tissue (Additional file 2: Table S11). Upon analysis using cord blood, 1916 CpGs (*P* < 5.8e−7) were associated with GA (Additional file 2: Table S12), 94 of these overlapped with the 4075 cord tissue GA-associated CpGs. Comparison of GA-associated vs. PTB-associated CpGs (Additional file 1: Figure S12) showed that > 95% of the 994 PTB-associated CpGs in cord tissue were also GA-associated (950 with *P* < 3.7e−7 and 993 with *P* < 1e−4 in an analysis using GA). Similarly, most of the 296 PTB-associated CpGs in cord blood remained GA-associated (284 with *P* < 5.8e−7 and 293 with *P* < 1e−4). These results suggests PTB-associated CpGs may also be a signature of GA. Gene ontology analyses performed on the 4075 cord tissue GA-associated CpGs and 1916 cord blood GA-associated CpGs gave similar conclusions as the analyses performed on PTB-associated CpGs. Specifically, cord tissue CpGs showed enrichment of pathways (Additional file 1: Figure S13) related to fetal growth and development (Additional file 1: Figure S14, Additional file 2: Table S13), while cord blood CpGs showed enrichment of immune response pathways (Additional file 1: Figure S15, Additional file 2: Table S14). We also compared these 1916 cord blood GA-associated CpGs with those reported by previous studies [7–12]. Of the 1916 cord blood GA-associated CpGs identified in the current study, 89% (1714 CpGs) could be replicated in at least 1 of the previous studies and 60% (1141 CpGs) in at least 2 of the previous studies (Additional file 1: Figure S16, Additional file 2: Table S15). However, the replication of the 296 cord blood PTB-associated CpGs with previous studies was relatively higher as > 95% of these CpGs replicated in at least 1 of the previous studies and > 80% replicated in at least 2 other studies.

## Discussion

In this study, we report associations with DNAm profiles in neonates born preterm by using tissues of different germinal origins, i.e., cord tissue and cord blood. The key findings from our study include (1) the replication of PTB/GA-associated cord blood CpGs across different studies and ethnicities to identify robust epigenetic signatures of PTB, (2) the identification of DNAm associations with PTB in cord tissue, and (3) the importance of evaluating the DNA methylomes of two germinally distinct neonatal tissues to capture a more comprehensive view of the molecular pathways associated with PTB.

### Replication of CpGs associated with GA/PTB in cord blood across different studies and ethnicities

More than 95% of the CpGs identified in our cord blood PTB EWAS were replicated in previous PTB/GA EWAS studies [7–12]. In particular, cg23062810 from *CLIP2* gene was replicated across six independent studies. *CLIP2* gene also seems to be a hotspot for PTB/GA-associated DNAm changes, as 6 additional CpGs have been previously reported from this gene—cg16356456 [7–12], cg04952324 [8–11], cg11573518 [11], cg02935052 [11], cg21375204 [11], and cg19501108 [10]. Notably, 2 CpGs adjoining cg23062810, i.e., cg16356456 and cg11573518, also showed moderate significance (*P* value < 10^−5^) in our study. The CpG trio of cg23062810, cg16356456, and cg11573518 is a promising candidate epigenetic signature for functional studies, as they are not only consistently reported to be hypermethylated in cord blood of preterm neonates, but also span a short 224-bp genomic region containing DNaseI hypersensitive site and several known transcription factor binding sites. CLIP2 is a cytoplasmic linker protein expressed in the brain [26], with its haploinsufficiency linked to motor coordination abnormalities [27]. *CLIP2* deletion is linked to Williams-Beuren syndrome, but deletion of a single copy alone is insufficient to result in the physical or cognitive characteristics of the disease [28].

Furthermore, in spite of the interrogation of PTB associations in an Asian population within our study, we achieved robust replication of 16 CpGs across all 6 earlier PTB/GA EWAS studies conducted in other populations of Caucasian/African American origin. These 16 CpGs span 12 genes, with 4 of these genes containing at least 2 PTB-associated CpGs in the current study. These genes include interleukin 21 receptor (*IL21R*), a key component of the adaptive immune system [29]; *NCOR2*, a relatively ubiquitously expressed repressor linked to a wide variety of biological processes including metabolism, inflammation, and circadian rhythm [30]; proline-rich 5 like (*PRR5L*), involved in the cellular response to oxidative stress [31]; and insulin-like growth factor 2 mRNA-binding protein 1 (*IGF2BP1*), a tightly regulated cell proliferation protein highly expressed during embryogenesis [32, 33]. Notably, the *PRR5L* gene carries 10 previously reported GA/PTB-associated CpGs, 3 of which we found to be PTB-associated in the current study (cg08943494, cg00220721, cg22117805). Although the exact function of PRR5L with respect to pregnancy is unknown, PRR5L suppresses a key regulator of cellular mTORC2 in vitro, which in turn is regulated by lysophosphatidic acid (LPA) and Gα12 activity [34]. LPA is implicated in the maintenance of pregnancy [35], uterine contractility [36], and infection-related preterm labor [37]; while Gα12 is a molecular regulator of extracellular stimuli, including oxidative stress [38]. There is also emerging evidence that mTOR-related genes are differentially expressed between term and preterm labor as well as between labor and non-labor myometrial [39].

### Identification of associations between DNAm and PTB in cord tissue

In addition to the findings from cord blood, we identified 994 CpGs to significantly associate with PTB in cord tissue, of which only 10 CpGs overlapped with cord blood CpGs. Our cord tissue findings provide new insights into the epigenetic landscape of neonates born preterm as this birth tissue has not been explored in this context before. Most importantly, the analysis of two neonatal tissues representing different cell type lineages provides a wider coverage of biological processes associated with PTB.

### Combination of EWAS in two neonatal tissues captures a comprehensive view of the molecular pathways associated with PTB

The two neonatal tissues gave deeper insights into the plausible molecular pathways associated with PTB. Gene networks in cord blood indicated the role of inflammation in PTB, which is in agreement with the previous findings implicating the role of inflammation in the etiology of PTB [6, 40]. The top most statistically significant PTB-CpGs from cord blood were found in genes involved in inflammation such as *TRAF5*, a key regulator of both canonical (via TNFα [41]) and non-canonical (via lymphotoxins [42]) NF-kappaB activation, and *MYLK*, a relatively ubiquitously expressed gene implicated in several inflammatory diseases [43] and also the main target for oxytocin-induced phosphorylation, downregulation of which follows uterine contraction at term [44]. Immune-related genes that were highly reproduced across different studies include *NCOR2*, an integral corepressor within the Notch signaling [45] with links to NK-kappaB-mediated apoptosis [46]; zinc finger and BTB domain containing 7B (*ZBTB7B*), a key regulator of CD4+T cell commitment [47]; PDZ and LIM domain protein 2 (*PDLIM2*), a key inhibitor of inflammatory response through NF-kappaB [48]; and *IL2RA*, a key component in immunological function primarily through the establishment of T cell immunological memory [49].

Gene ontology terms linked with cord blood CpGs also reflected the dominance of immune-related biological processes despite the adjustment for cellular heterogeneity. The largest gene ontology cluster enriched in the pathway analysis was regulation of T cell differentiation, a hallmark of innate immune system development, which includes genes such as tripartite motif containing 22 (*TRIM22*, interferon signaling [50]), interleukin 1 receptor-associated kinase 2 (*IRAK2*, inflammatory response to infection [51]), and caspase recruitment domain family member 11 (*CARD11*, critical component of T cell and B cell signaling [52]). The next two largest clusters also featured several gene ontology terms with various immune-related nuclear factor kappa-light-chain-enhancer of activated B cells (NF-kappaB) components.

The role of immune-related genes is also apparent, albeit to a smaller degree, in cord tissue CpGs significantly associated with PTB. Prominent examples include *NKFBIA*, which binds to the nuclear localization signal of the inflammatory response element NF-kappa-B/REL complex, preventing transcription and inflammatory response [53]; and *NFIL3* (nuclear factor, interleukin 3 activated), a transcription regulator, mostly inhibiting many genes [54], but also known to activate interleukin-3 [55], mediating pro-B lymphocyte survival [56]. Incidentally, NFKBIA appears to be upregulated in placenta with history of chorioamnionitis, as well as those complicated by preterm premature rupture of membrane (PPROM) cases [57]. Upregulation of NFKBIA is suggested to be a form of anti-inflammatory response to inflammatory insults [57]. NFIL3, on the other hand, is downregulated with term within CD34+ cord blood fractions [58], consistent with the comparatively larger population of immature hematopoietic progenitor population in preterm cord blood. Collectively, differentially methylated CpGs from both tissues highlight the role of inflammatory genes in PTB, with a larger representation in cord blood than cord tissue.

Cord tissue CpGs significantly associated with PTB were found in genes with more diverse gene functions as opposed to primarily immune responses seen in cord blood. These included general transcription factor genes such as protein C-Ets-2 (*ETS2*) [59] and specificity protein 1 (*SP1*) [60]. Incidentally, ETS2 was previously reported to be downregulated in preterm placentas with spontaneous labor [61], while differentially expressed genes with respect to peripheral blood in mothers who delivered preterm possessed over-representation of SP1 binding sites within their promoters [62]. Gene ontology enrichment analysis revealed cord tissue CpGs to mostly lie in genes related to physiological growth and development. In particular, bone development was the largest grouped cord tissue gene ontology result, including genes such as parathyroid hormone 1 receptor (*PTH1R*, surface receptor of osteoblasts [63]), bone morphogenetic proteins 2 and 6 (*BMP2*, *BMP6*, simulator of bone growth [64]), and matrix metallopeptidase 7 (*MMP7*, associated with bone remodeling [65]). This was followed by regulation of Wnt signaling pathway—a pathway which plays a central role in embryonic development [66], with members such as Wnt family members 8A and 11 (*WNT8A*, involved in axis patterning [67]; *WNT11* is involved in the skeletal, kidney, and lung development [68]). The third largest gene cluster was related to extracellular matrix (ECM) organization. ECM impacts a number of cellular functions critical for normal fetal development and morphogenesis [69]. The most familiar developmental function attributed to ECM is cell migration during fetal development and organogenesis that is facilitated by cycles of cell adhesion and deadhesion. ECM also plays important structural roles in defining tissue boundaries, branching morphogenesis, developing tissue asymmetry and growth factor signaling.

### Study limitations

This study has a few limitations. First, while we have observed global DNAm alterations in the two neonatal tissues at CpG sites assayed using the Infinium HumanMethylation450 platform, the CpGs assayed by the platform were not randomly selected from the DNA methylome. Consequently, it is unclear how the findings will extend to the rest of the DNA methylome. Second, as supported by our findings here and in a previous publication [23], EWAS conducted using different tissues can give very distinct findings. While use of clinically available tissues like cord tissue and cord blood is convenient, use of these two tissues may not completely mirror the effects of PTB in target tissues. Thus, further research is necessary to investigate if these findings can be extrapolated to the relevant tissues of interest. Third, while we have successfully replicated our findings in cord blood using results from published literature, due to the lack of availability of cord tissue DNAm data, we are unable to replicate the cord tissue findings in an independent cohort. However, the robust (> 95% CpGs) replication of the findings in previously reported PTB studies in cord blood and the use of larger sample size suggest that our novel cord tissue findings are likely to be robust too.

## Conclusion

Using DNAm profiles from two different neonatal tissues (cord tissue and cord blood), we provide the epigenetic status of a broader spectrum of molecular pathways associated with PTB. Our findings suggest that genes involved in inflammation and fetal developmental processes play a key role in PTB. Further research is necessary to identify the specific role played by these epigenetic changes on the postnatal developmental and health trajectories of the offspring.

## Methods

### Study population

Between June 2009 and September 2010, healthy pregnant women were recruited in their first trimester of pregnancy from two major public hospitals in Singapore, namely the KK Women’s and Children’s Hospital (KKH) and the National University Hospital (NUH), to participate in the Growing Up in Singapore Towards Healthy Outcomes (GUSTO) birth cohort study [70]. To participate in the study, pregnant women had to satisfy the following inclusion criteria: (1) be of at least 18 years of age; (2) hold Singapore citizenship or permanent residency, or intent to reside in Singapore for the next 5 years; (3) be of Chinese, Malay, or Indian ethnic origin, confirmed through homogeneous parental ethnic background and genotyping; (4) intent to deliver at either NUH or KKH; and (5) intent to donate cord tissue and cord blood. The exclusion criteria included (1) women on chemotherapy, (2) women with significant health conditions such as type 1 diabetes mellitus and psychosis, and (3) women on specific medications such as psychotropic drugs. The present analysis was restricted to live singleton births with infant DNAm data (cord tissue or cord blood).

### Determining GA, infant sex, and ethnicity

GA was determined by ultrasonography in the first trimester of pregnancy. PTB was defined as GA < 37 weeks. Child sex was extracted from the medical records. Ethnicity was self-reported by the mother at study recruitment.

### Tissue collection and processing

Detailed information on cord tissue and cord blood collection as well as processing has been previously described [23]. Briefly, cord blood was collected post-delivery by either dripping the blood in EDTA tubes for normal deliveries or collecting via a syringe in the event of assisted deliveries. Collected cord blood was centrifuged at 4 °C, 3000*g* for 5 min, and the buffy coat extracted was stored at − 80 °C until subsequent DNA extraction. DNA extraction of cord blood was carried out using QIAsymphony DNA Kit as per the manufacturer’s instructions. After collection of the cord blood, cord tissue was cleaned with phosphate buffer saline (PBS) solution. The cord was then snap-frozen in liquid nitrogen and stored at − 80 °C until subsequent DNA extraction. Before DNA extraction, frozen umbilical cords were crushed using a mortar and pestle, treated with 10 U/mL hydraluronidase enzyme and homogenized using a Xiril Dispomix Homogeniser. Proteinase K was added to the homogenate and incubated overnight at 55 °C. Cord tissue DNA was then extracted as described earlier [23].

### DNAm profiling and data processing

DNA methylomes for cord tissue and cord blood were profiled and processed separately using the Infinium HumanMethylation450 platform (Additional files 3 and 4). Data processing was conducted using an in-house quality control procedure that was previously described [71]. Briefly, we exported raw DNAm beta values from GenomeStudio™ and set probes with less than three beads for either the methylated or unmethylated channel or with detection *P* value > 0.01 to missing. We then performed color adjustment and normalization of the type 1 and 2 probes and excluded sex chromosome probes. As part of the study design for DNAm profiling, samples were randomized across chip and position on chip with respect to key variables including GA, infant sex, and ethnicity. Thus, expectedly, PTB did not associate with chip or position effects. For both tissues, a principal component analysis of the raw DNAm revealed chip to associate most significantly with the raw DNAm data. DNAm data for both tissues were thus adjusted for chip using COMBAT, removing CpGs with missing values across all 12 positions on any chip [72]. For the remainder technical variables that were associated with top principal components of the DNAm data, but were not randomized, PTB was associated with bisulfite conversion batch (both cord tissue and cord blood) and DNA extraction batch (cord tissue only), and these variables were included as covariates in all regression models. Finally, cross-hybridizing probes [73, 74], CpGs on or within a single-base extension of a SNP and CpGs with multi-modal distributions were excluded from the analysis. As CpGs with low inter-individual variation in each tissue may be more reflective of the technical variation than true biological signal, to reduce false positives and increase overall study power [75, 76], we further excluded CpGs that had low inter-individual variation in each tissue (i.e., DNAm range under 10% or DNAm of the 99th centile minus 1st centile under 5%). After quality control and exclusion of CpGs with low variability, 134,676 CpGs (cord tissue) and 85,624 CpGs (cord blood) were available for subsequent analysis. For infant cord tissue, cellular proportions for stromal, endothelial, epithelial, and blood were estimated using a reference panel [77] and their principal components were adjusted as covariates in all regression models. Likewise, for infant cord blood, cell-type proportions for granulocytes, monocytes, natural killer cells, B cells, CD4+ T cells and CD8+ T cells were estimated using a reference panel [77] and their principal components were adjusted as covariates in all regression models. CpGs were annotated with respect to gene features (promoter, 5′-UTR, exon, intron, 3′-UTR, TTS, and intergenic regions) using Homer *annotatePeaks* function (hg19).

### Statistical analysis

#### Association between DNAm and PTB

To examine the association between DNAm and PTB for each tissue, we fitted a linear regression model with DNAm as the dependent variable and PTB as the independent variable, adjusted for technical variables that associated with PTB (bisulfite conversion batch and DNA extraction batch), infant sex, ethnicity, and estimated cell-type proportions. Infant sex and ethnicity were selected as covariates for inclusion in the regression models based on a priori evidence of their playing key roles in DNAm and/or PTB. For each CpG, individuals with outlier DNAm values (defined as DNAm values exceeding the cohort median ± twice the interquartile range for each CpG) were excluded from the analysis. PTB was coded as a binary variable, with 1 = term and 0 = preterm; thus, a negative regression coefficient implies that DNAm levels were generally higher among the preterm infants compared to term infants.

For CpGs significantly associated with PTB in at least one tissue, we also assessed whether there was evidence of tissue-dependent effects. This analysis was performed by fitting a general linear model with an unstructured covariance structure to a combined dataset with DNAm data from both tissues, including main effect terms for tissue and PTB, and an interaction term between PTB and tissue and other covariates. The interaction term between PTB and tissue provides an estimate of the difference in PTB-DNAm association in the two tissues, and a statistical test of this interaction term provides a formal test of tissue-dependent effects.

#### Pathway analysis

For genes where the CpGs were significantly associated with PTB after adjustment for multiple testing using a Bonferroni correction, we further examined them for enrichment of gene ontology biological pathways using the *gometh* function in the MissMethyl R package [78], which maps CpG sites to their nearest gene and corrects for bias due to non-uniform coverage of genes on the Infinium HumanMethylation450 array. To consolidate and summarize the pathway enrichment analysis results from *gometh*, nominally significant GO terms (*P* < 0.01) within the “biological processes” category were further run through the REVIGO tool, which avoids reporting GO terms with greater than 70% in semantic similarity measure [79]. As GO terms involving many genes may not inform precise gene functionalities, larger GO terms (containing 300 or more genes) were removed before running REVIGO. The results from REVIGO were visualized using TreeMaps.

#### Sensitivity analysis

We also conducted sensitivity analyses where we further adjusted for mode of delivery, maternal hypertension, maternal age, smoking, parity, and position on chip (sensitivity analysis 1). To further allow for the possibility of unmeasured technical artifacts or un-accounted cell-type proportions, we also used surrogate variable analysis (SVA) to directly estimate sources of batch effects and/or cell-type composition from the DNAm data. The resulting estimated surrogate variables from the SVA could potentially capture both batch effects and cell-type composition. We conducted additional sensitivity analyses (sensitivity analysis 2), where we repeated the association analyses between PTB and DNAm, adjusting for surrogate variables from the SVA, on top of infant sex and ethnicity [80, 81].

#### Comparison of PTB-associated CpGs in cord blood with previously published studies

We compared our cord blood PTB EWAS findings with PTB/GA EWAS findings from previous studies [7–12]. For a fair comparison, we restricted this analysis to the studies conducted using the same Infinium HumanMethylation450 platform. We also applied the DNAm GA clocks published by Knight et al. [24] and Bohlin et al. [11] to predict GA in our study samples. The clocks published by Knight et al. and Bohlin et al. were applied to our cord tissue and cord blood DNAm data separately. For this analysis, raw DNAm data without any processing or quality control filtering was used.

#### Associations between DNAm and GA

Since a number of previous EWAS were conducted using GA as a continuous variable instead of PTB as a binary variable, we also conducted an additional analysis using GA as a continuous variable. For each tissue, we fitted a linear regression model with DNAm as the dependent variable and GA as the independent variable, adjusted for the same covariates as before. Pathway analysis and comparison with earlier reports were performed similarly.

## Additional files

Additional file 1: Supplementary figures. (PDF 6638 kb)

Additional file 2: Supplementary tables. (ZIP 3966 kb)

Additional file 3: DNA methylation data for cord blood. (PHENO 2410 kb)

Additional file 4: DNA methylation data for cord tissue. (PHENO 1130000 kb)

## Abbreviations

DNAm: DNA methylation
EWAS: Epigenome-wide association study
GA: Gestational age
GUSTO: Growing Up in Singapore Towards Healthy Outcomes
KKH: KK Women’s and Children’s Hospital, Singapore
NUH: National University Hospital, Singapore
PTB: Preterm birth
SVA: Surrogate variable analysis
