# Supplementary material for: Analysis of two birth tissues provides new insights into the epigenetic landscape of neonates born preterm
Source: Clin Epigenetics. 2019 Feb 11;11:26. doi: 10.1186/s13148-018-0599-4 (PMC6371604; doi:10.1186/s13148-018-0599-4)
Supplement: Supplementary file 1 — Supplementary figures. (PDF 6638 kb) [file 13148_2018_599_MOESM1_ESM.pdf]

Supplementary Figures

Version 17
Last updated: October 19, 2018

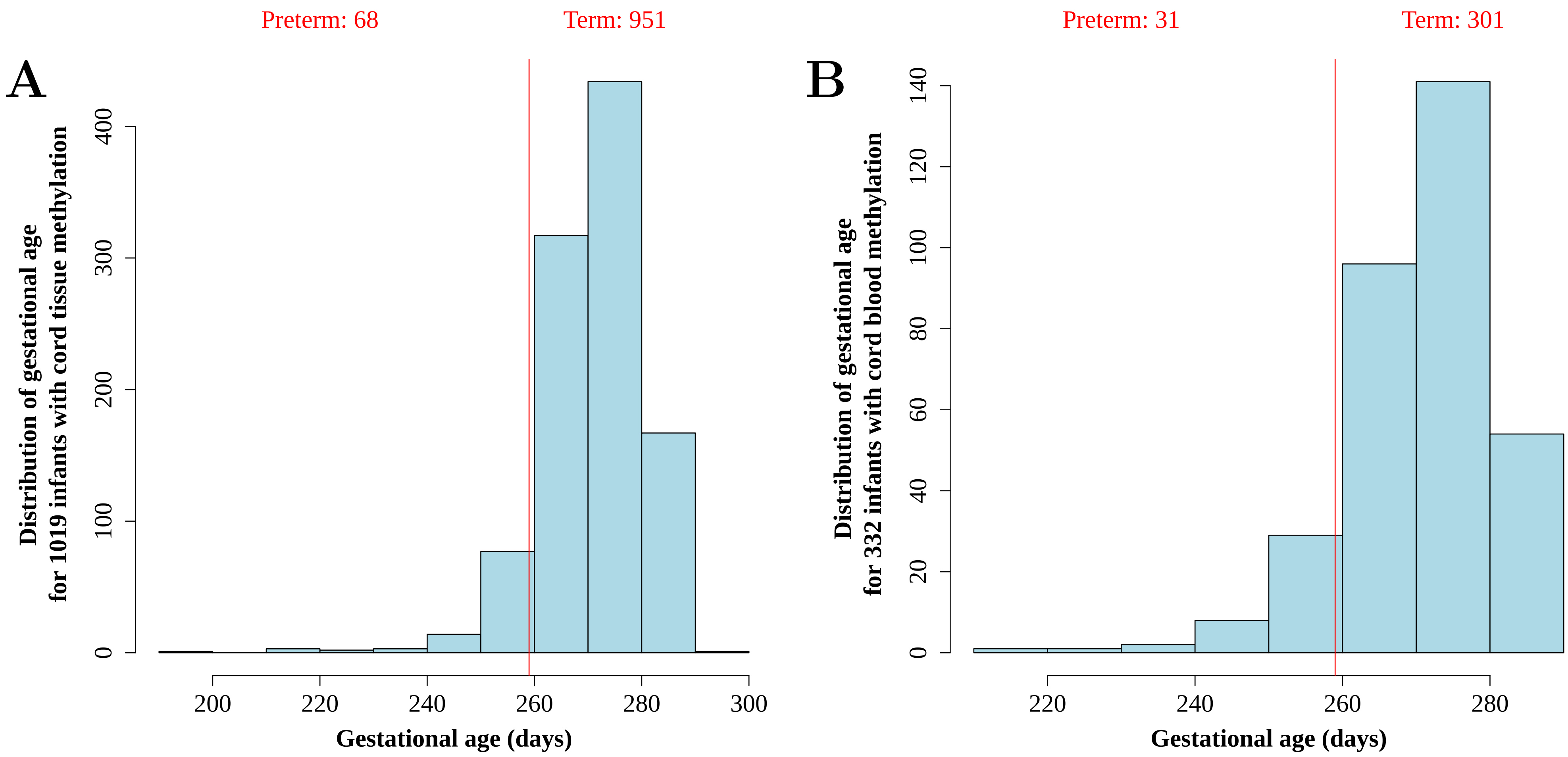

Supplementary Figure 1. Gestational age distribution of subjects with respect to infant tissue type. This study utilized 1,019 infant cord tissue samples and 332 infant cord blood samples. The gestational age distribution of infant cord tissue samples (**A**) and cord blood samples (**B**) are represented by corresponding histograms with the horizontal axis reflecting gestational age in days and the vertical axis reflecting the number of subjects.

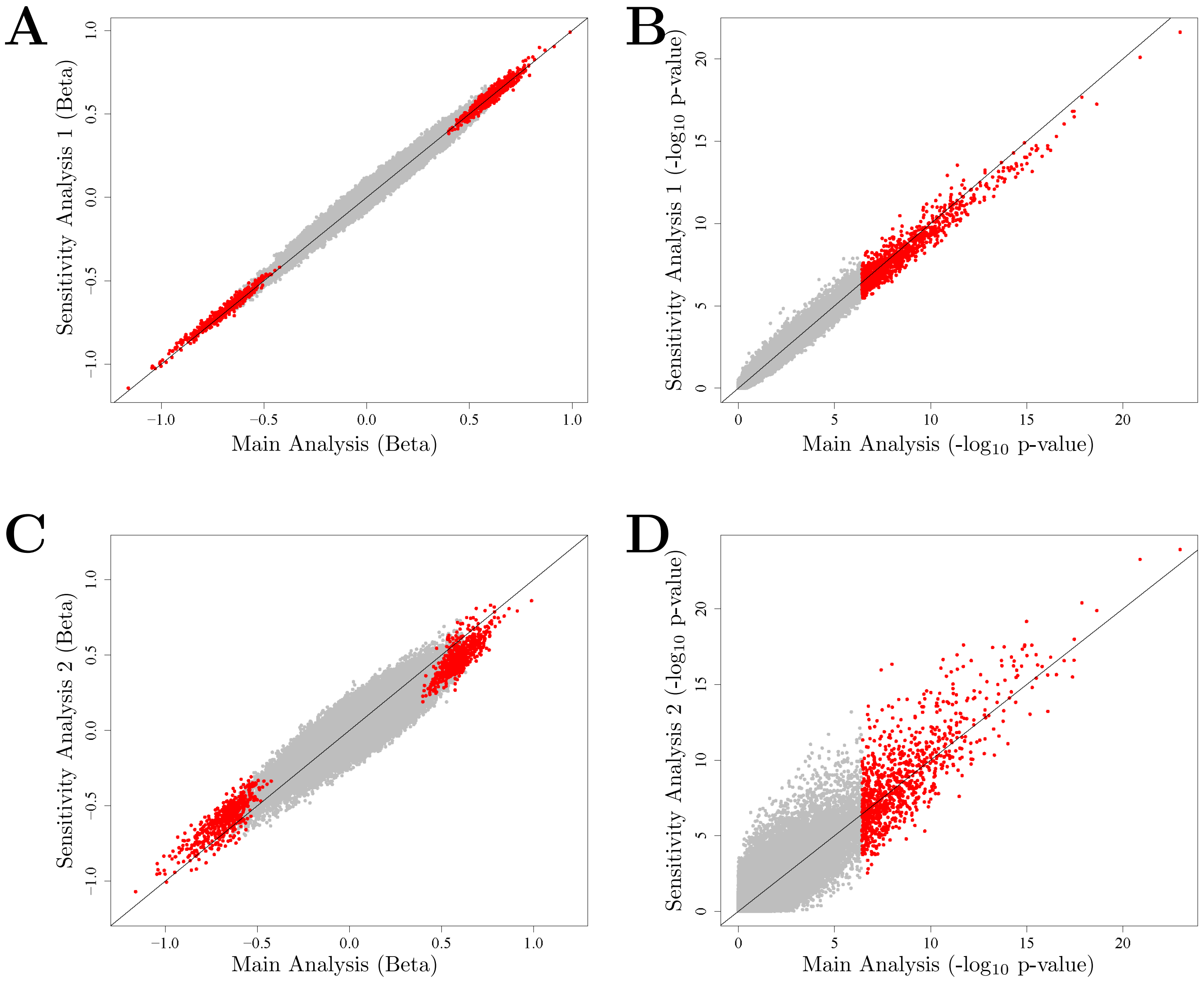

Supplementary Figure 2. Sensitivity analysis of cord tissue DNA methylation levels and preterm births. Scatterplots comparing the change in DNA methylation Z-score (denoted as "Beta" in **A, C**) and negative $\log_{10}$ p-values (**B, D**) between the main analysis (cord tissue methylation with respect to preterm birth status, adjusted for infant sex, ethnicity, cell-type proportions, bisulfite conversion batch, and DNA extraction batch) on the horizontal axis of **A-D**, sensitivity analysis 1 (cord tissue methylation with respect to preterm birth status, adjusted for infant sex, ethnicity, cell-type proportions, bisulfite conversion batch, DNA extraction batch, maternal age, maternal history of hypertension treatment, maternal exposure to cigarette smoke, mode of delivery, infant firstborn status, and chip position) on the vertical axis of **A-B**, and sensitivity analysis 2 (Surrogate Variable Analysis) on the vertical axis of **C-D**. Of the 994 CpGs Bonferroni significant in the main analysis (indicated as red dots in the scatterplot, in contrast with the grey dots which were not Bonferroni significant in the main analysis), 869 (87%) remained Bonferroni significant and all (100%) reflecting nominal significances (p-value $< 10^{-4}$) in sensitivity analysis 1, while 790 (79%) of them remained Bonferroni significant and 980 (99%) reflecting nominal significances (p-value $< 10^{-4}$) in sensitivity analysis 2. The diagonal line across all four scatterplots represents y=x.

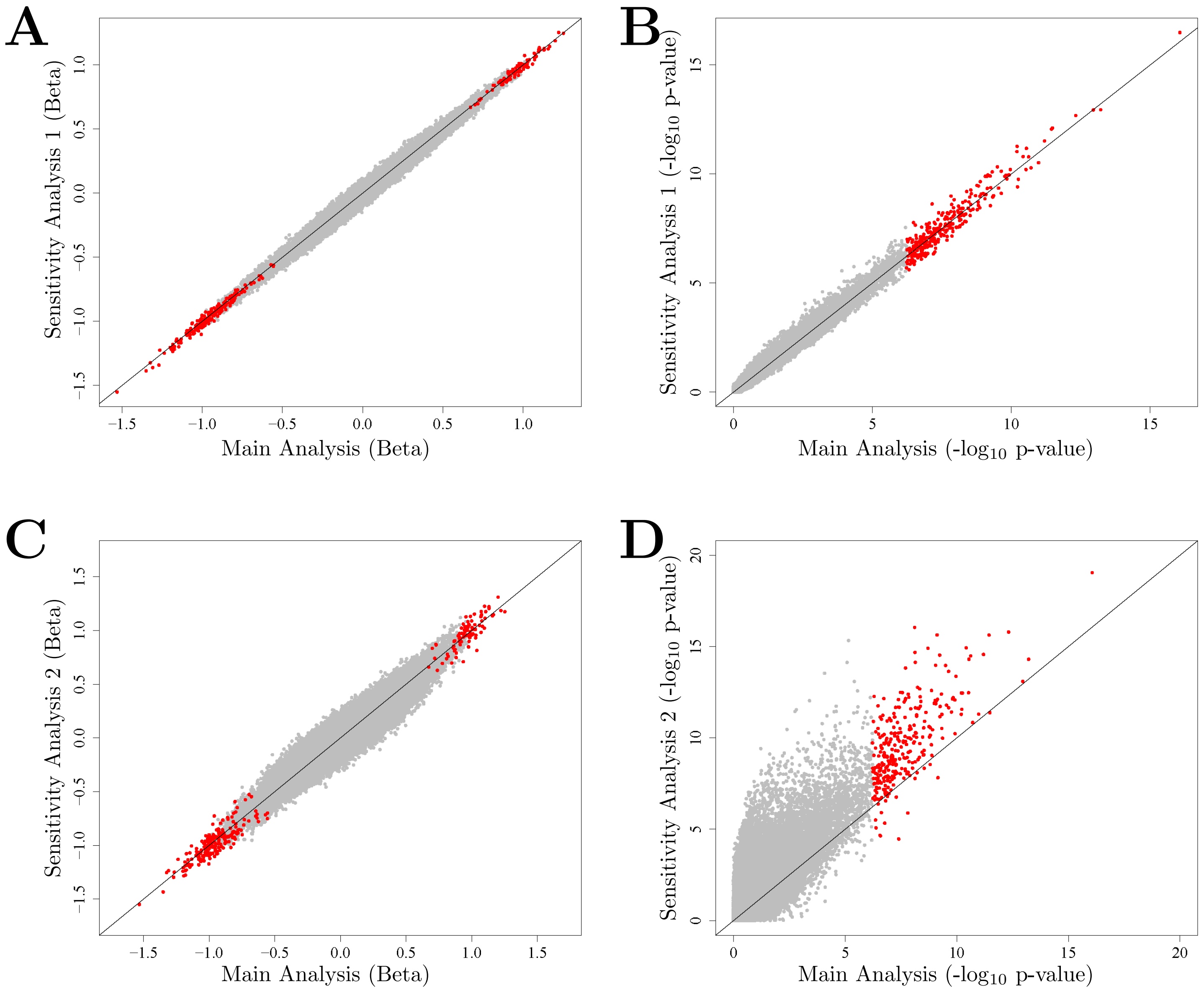

Supplementary Figure 3. Sensitivity analysis of cord blood DNA methylation levels and preterm births. Scatterplots comparing the change in DNA methylation Z-score (denoted as "Beta" in **A, C**) and negative $\log_{10}$ p-values (**B, D**) between the main analysis (cord blood methylation with respect to preterm birth status, adjusted for infant sex, ethnicity, cell-type proportions, and bisulfite conversion batch) on the horizontal axis of **A-D**, sensitivity analysis 1 (cord blood methylation with respect to preterm birth status, adjusted for infant sex, ethnicity, cell-type proportions, bisulfite conversion batch, maternal age, maternal history of hypertension treatment, maternal exposure to cigarette smoke, mode of delivery, infant firstborn status, and chip position) on the vertical axis of **A-B**, and sensitivity analysis 2 (Surrogate Variable Analysis) on the vertical axis of **C-D**. Of the 296 CpGs Bonferroni significant in the main analysis (indicated as red dots in the scatterplot, in contrast with the grey dots which were not Bonferroni significant in the main analysis), 277 (94%) remained Bonferroni significant and all (100%) reflecting nominal significances (p-value $< 10^{-4}$) in the sensitivity analysis 1, while 288 (97%) of them remained Bonferroni significant and all (100%) reflecting nominal significances (p-value $< 10^{-4}$) in sensitivity analysis 2. The diagonal line across all four scatterplots represents y=x.

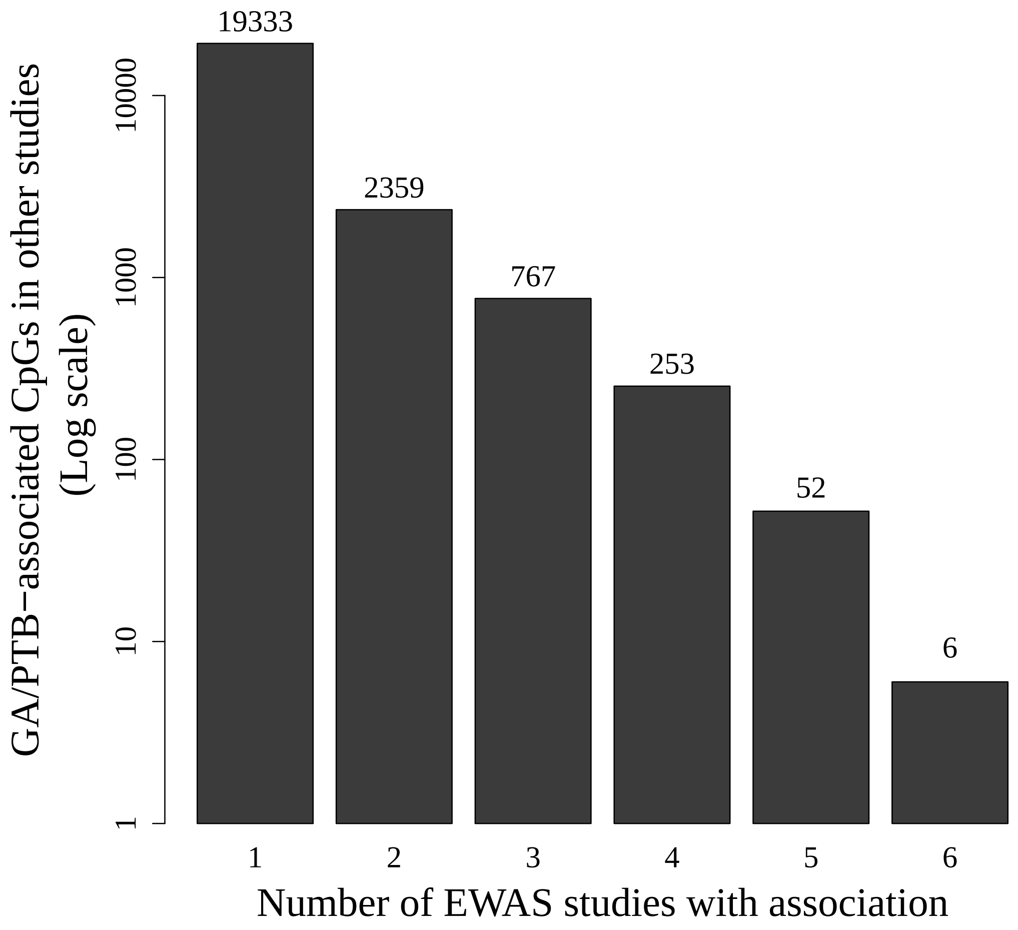

Supplementary Figure 4. Cord Blood CpGs reported to associate with gestational age (GA) or preterm birth (PTB) in earlier studies, but not PTB-associated in the current study. We compiled 23,066 cord blood CpGs previously found to be associated with GA or PTB in six studies, of which 22,770 CpGs were not found to be PTB-associated in the current study (**Figure 3**). This histogram catalogues the reproducibility of these 22,770 CpGs within these six studies.

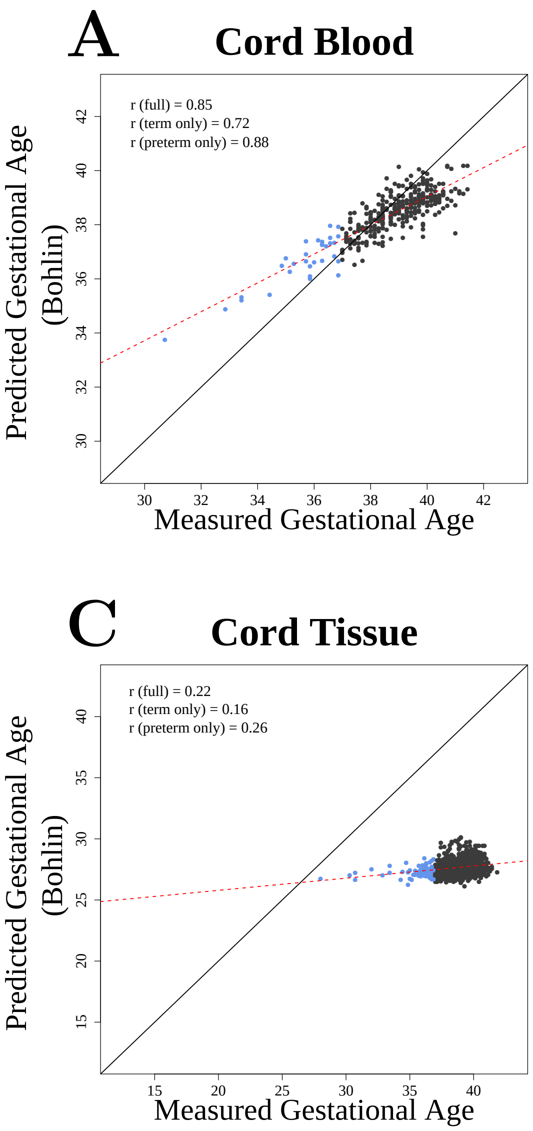

Supplementary Figure 5. Scatterplots of predicted vs measured gestational ages (GA) based on cord blood and cord tissue CpGs. Predicted GA was calculated using the epigenetic clocks developed by Bohlin *et al.* (2016) and Knight *et al.* (2016) on cord blood (**A, B**) and cord tissue (**C, D**) CpGs respectively. These predicted GA (in weeks, vertical axis) are plotted against the measured GA (in weeks, horizontal axis). Plots for each method on cord blood/tissue is presented with three Pearson correlation coefficients (r): full – correlation across all measured GA against corresponding predicted GA, term only – correlation on measured GA 37 weeks and above against corresponding predicted GA, and preterm only – correlation on measured GA under 37 weeks against corresponding predicted GA. The red dotted line represents the regression line between measured and predicted GA and the black line represents $x = y$.

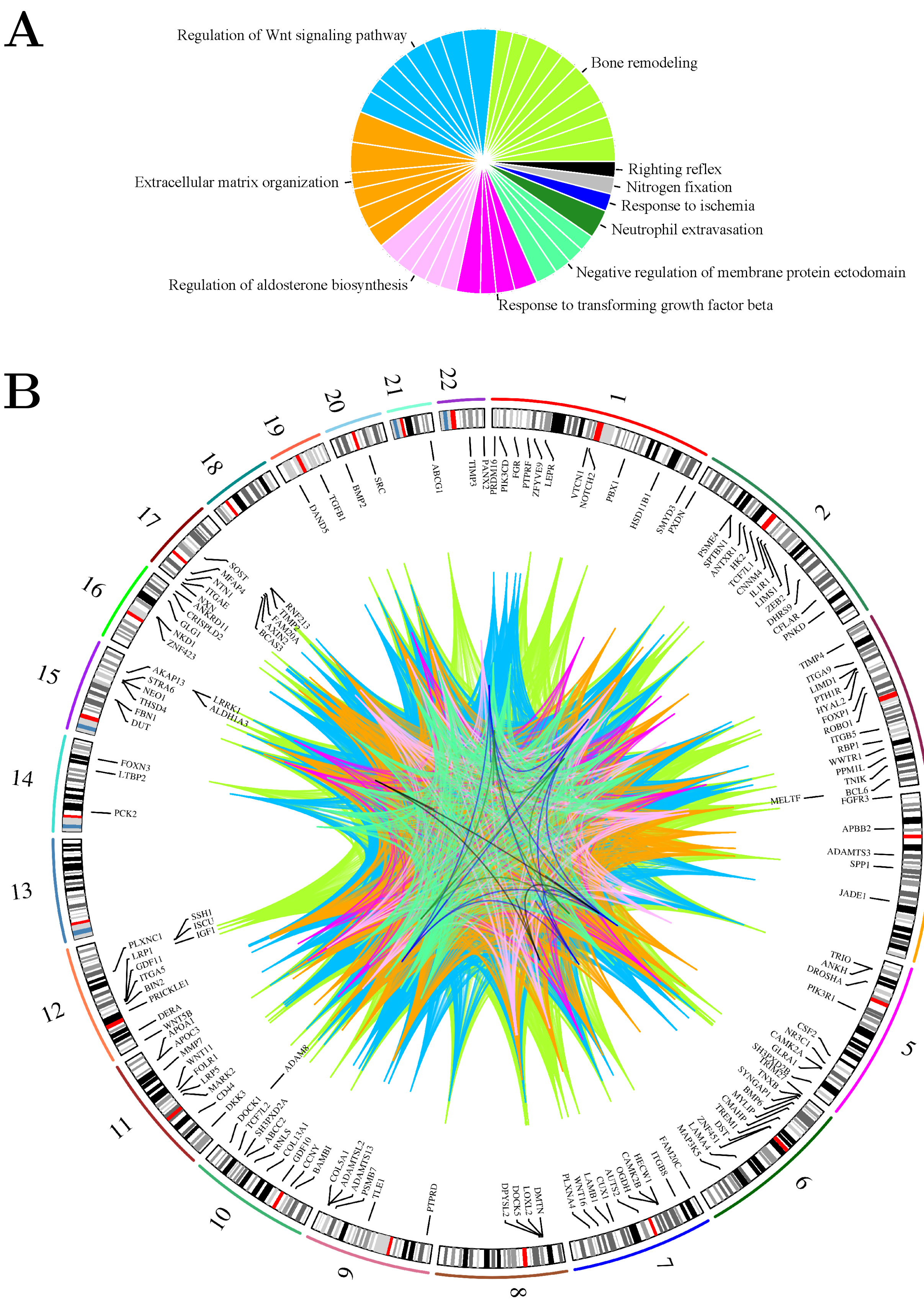

Supplementary Figure 6. Preterm birth (PTB)-associated cord tissue CpGs lie in genes linked to growth and development. Gene ontology (GO) enrichment was performed on genes containing any of the 994 significantly associated infant cord tissue CpGs with respect to PTB using missMethyl. 149 GO terms relating to biological processes (parent GO term containing under 300 genes, $p < 0.01$) were then consolidated using REVIGO (semantic similarity measure between each GO term < 0.7) resulting in 10 GO clusters from 41 unique GO terms encompassing 146 genes. The reclassified enriched GO clusters are represented in the pie chart **A**, with each slice representing one GO term. The size of each slice represents the absolute $\log_{10}$ p-value of the corresponding GO term. The Circos plot (**B**) represents the 146 genes containing significantly associated infant cord tissue CpGs themselves, linked with respect to their presence within the clusters as defined in **A**. “Nitrogen fixation” only had one gene containing a significantly associated infant cord tissue CpG and hence does not contain gene linkers represented in **B**.

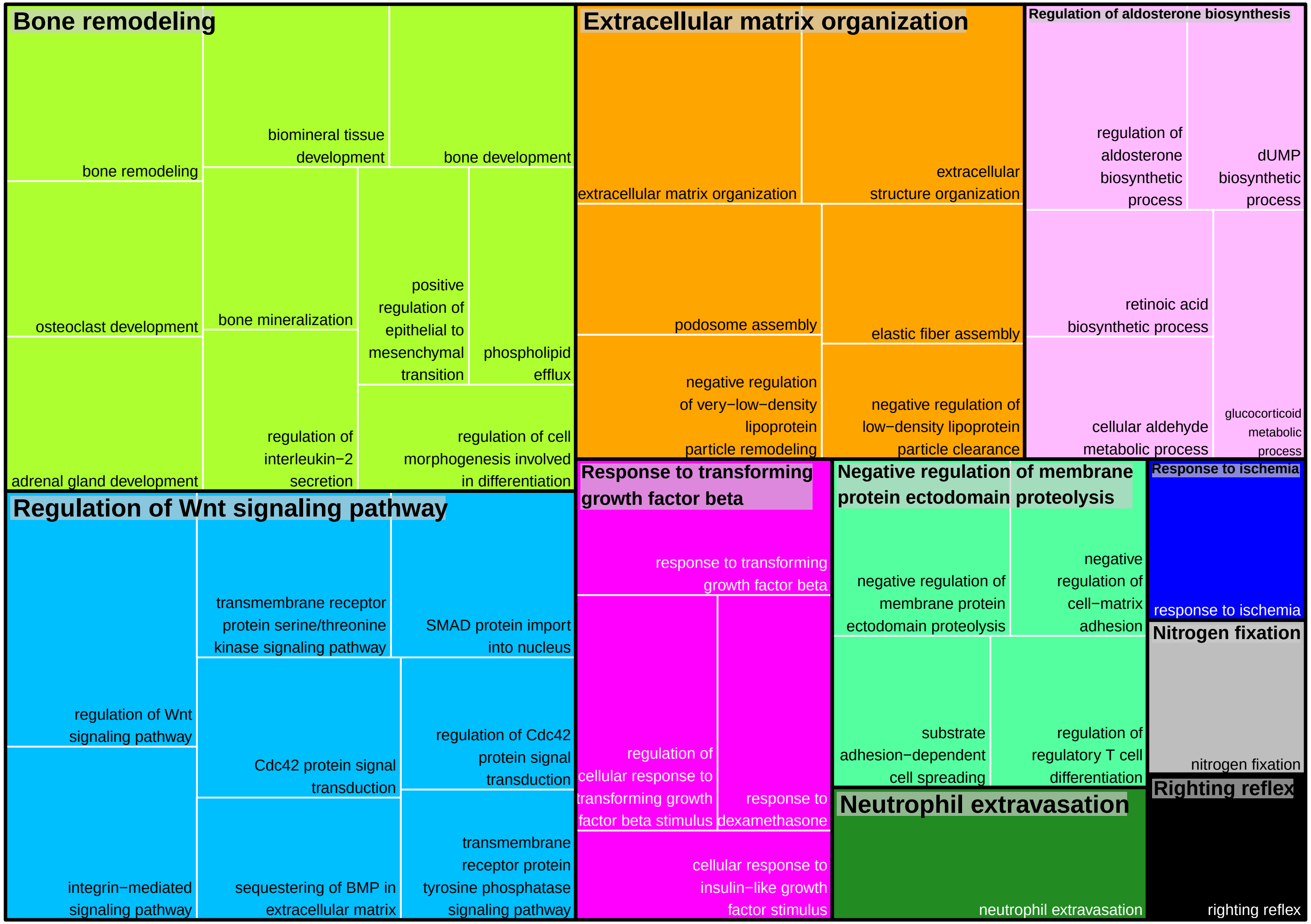

Supplementary Figure 7. REVIGO treemap of enriched gene ontologies from Preterm birth (PTB)-associated cord tissue CpGs. Gene ontology (GO) enrichment was performed on genes containing any of the 994 significantly associated infant cord tissue CpGs, against genes lying in any of the 134,676 cord tissue CpGs analyzed as the background, with respect to PTB using missMethyl. REVIGO was subsequently used to reclassify the biological process-related enriched GO terms (parent GO term containing under 300 genes, semantic similarity measure between each GO term < 0.7) resulting in 10 GO clusters from 41 unique GO terms. Each GO cluster is represented with a unique color with the cluster name on the top left corner enclosed by bold black outlines in the treemap while the individual GO terms are expounded on the bottom right corner enclosed by white outlines. The size of each treemap cell was automatically generated by REVIGO based on the statistical significances of the GO terms.

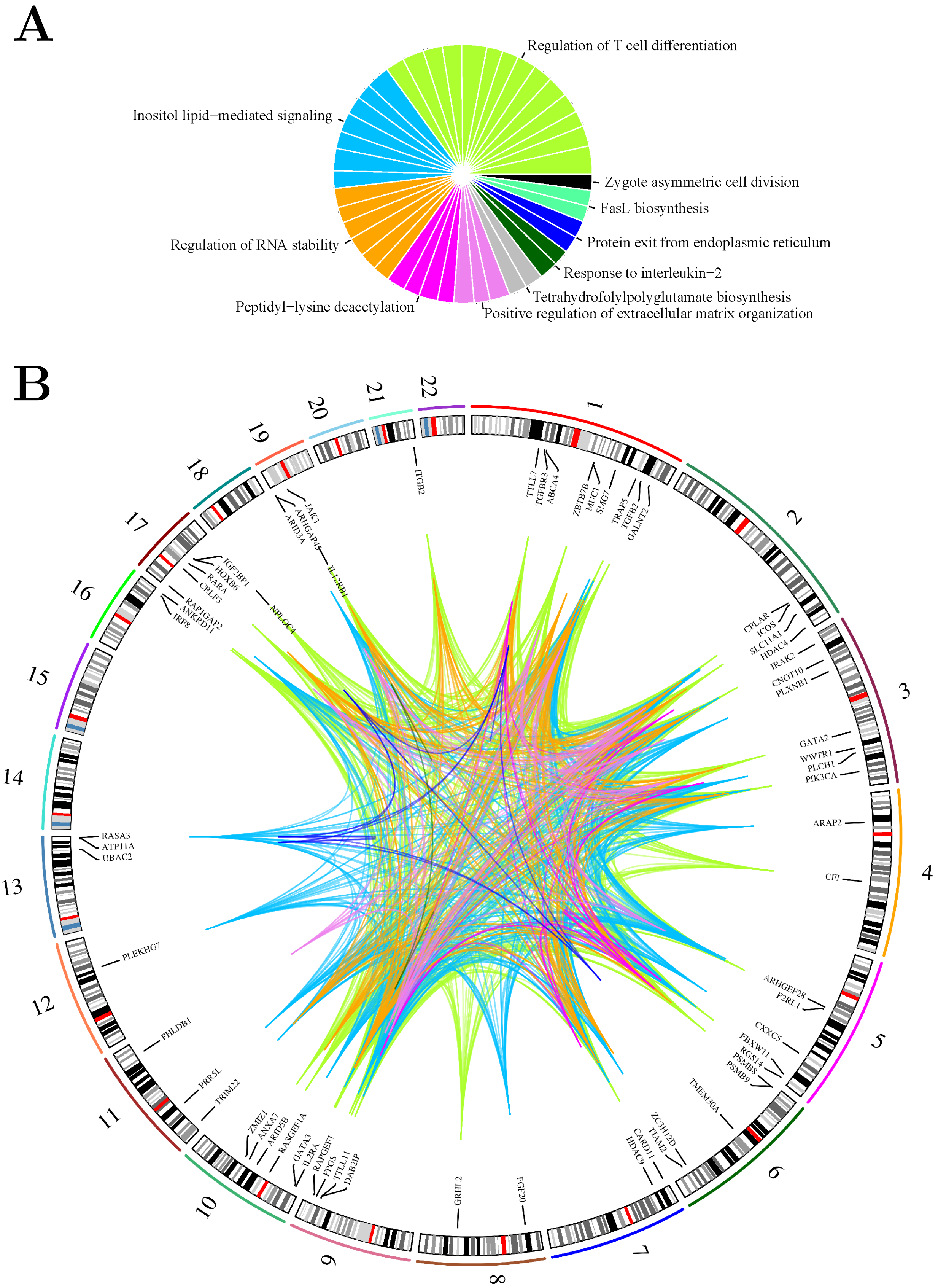

Supplementary Figure 8. Preterm birth (PTB)-associated cord blood CpGs lie in genes linked to immune response. Gene ontology (GO) enrichment was performed on genes containing any of the 296 significantly associated infant cord blood CpGs with respect to PTB using missMethyl. 170 GO terms relating to biological processes (parent GO term containing under 300 genes, $p < 0.01$) were then consolidated using REVIGO (semantic similarity measure between each GO term < 0.7) resulting in 10 GO clusters from 43 unique GO terms encompassing 66 genes. The reclassified enriched GO clusters are represented in the pie chart **A**, with each slice representing one GO term. The size of each slice represents the absolute $\log_{10}$ p-value of the corresponding GO term. The Circos plot (**B**) represents the 66 genes containing significantly associated infant cord blood CpGs themselves, linked with respect to their presence within the clusters as defined in **A**. "FasL biosynthesis", "Tetrahydrofolylpolyglutamate biosynthesis", and "zygote asymmetric cell division" only had one gene containing a significantly associated infant cord blood CpG and hence do not contain gene linkers represented in **B**.

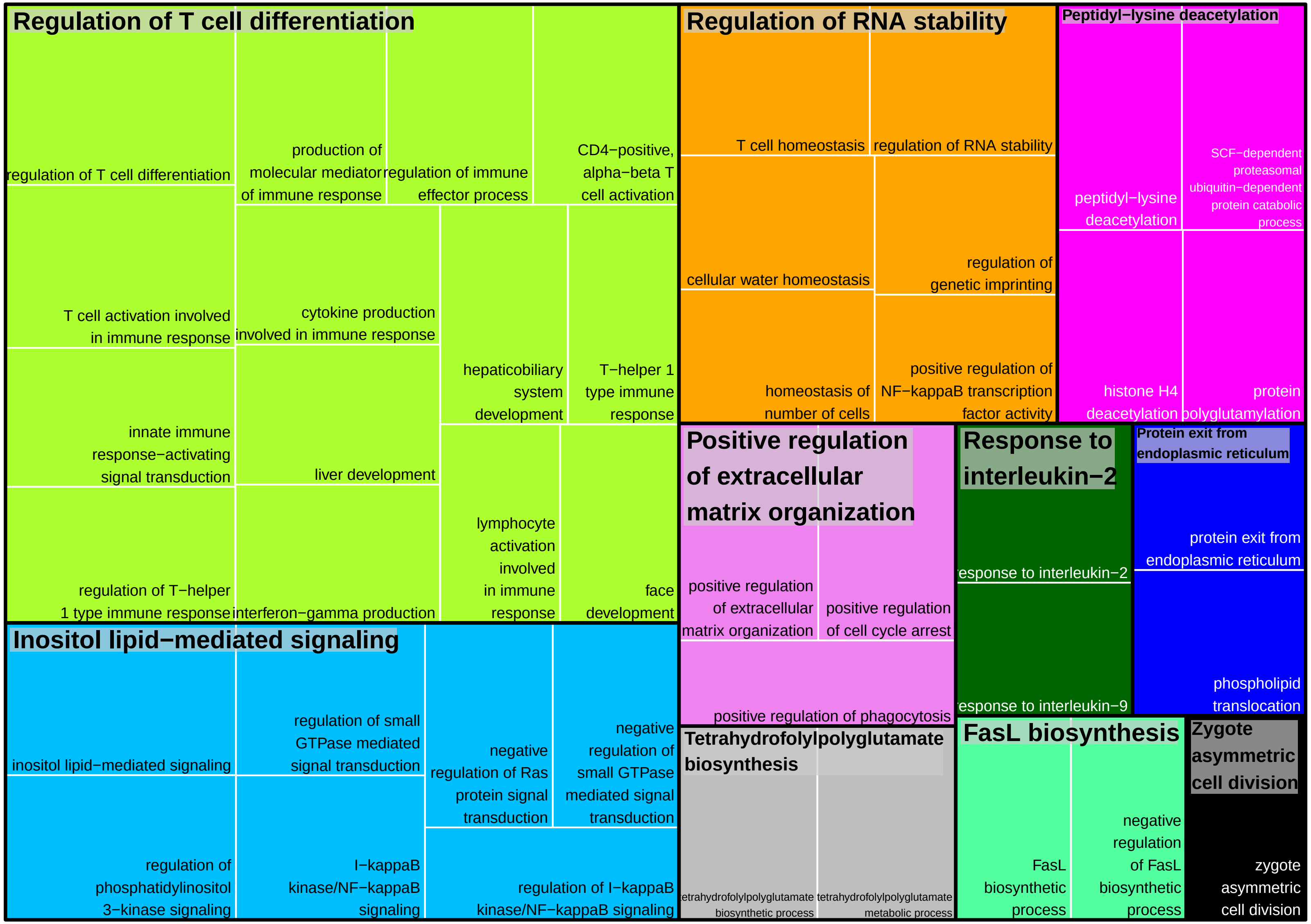

Supplementary Figure 9. REVIGO treemap of enriched gene ontologies from Preterm birth (PTB)-associated cord blood CpGs. Gene ontology (GO) enrichment was performed on genes containing any of the 296 significantly associated infant cord blood CpGs, against genes lying in any of the 85,624 cord blood CpGs analyzed as the background, with respect to PTB using missMethyl. REVIGO was subsequently used to reclassify the biological process-related enriched GO terms (parent GO term containing under 300 genes, semantic similarity measure between each GO term < 0.7) resulting in 10 GO clusters from 43 unique GO terms. Each GO cluster is represented with a unique color with the cluster name on the top left corner enclosed by bold black outlines in the treemap while the individual GO terms are expounded on the bottom right corner enclosed by white outlines. The size of each treemap cell was automatically generated by REVIGO based on the statistical significances of the GO terms.

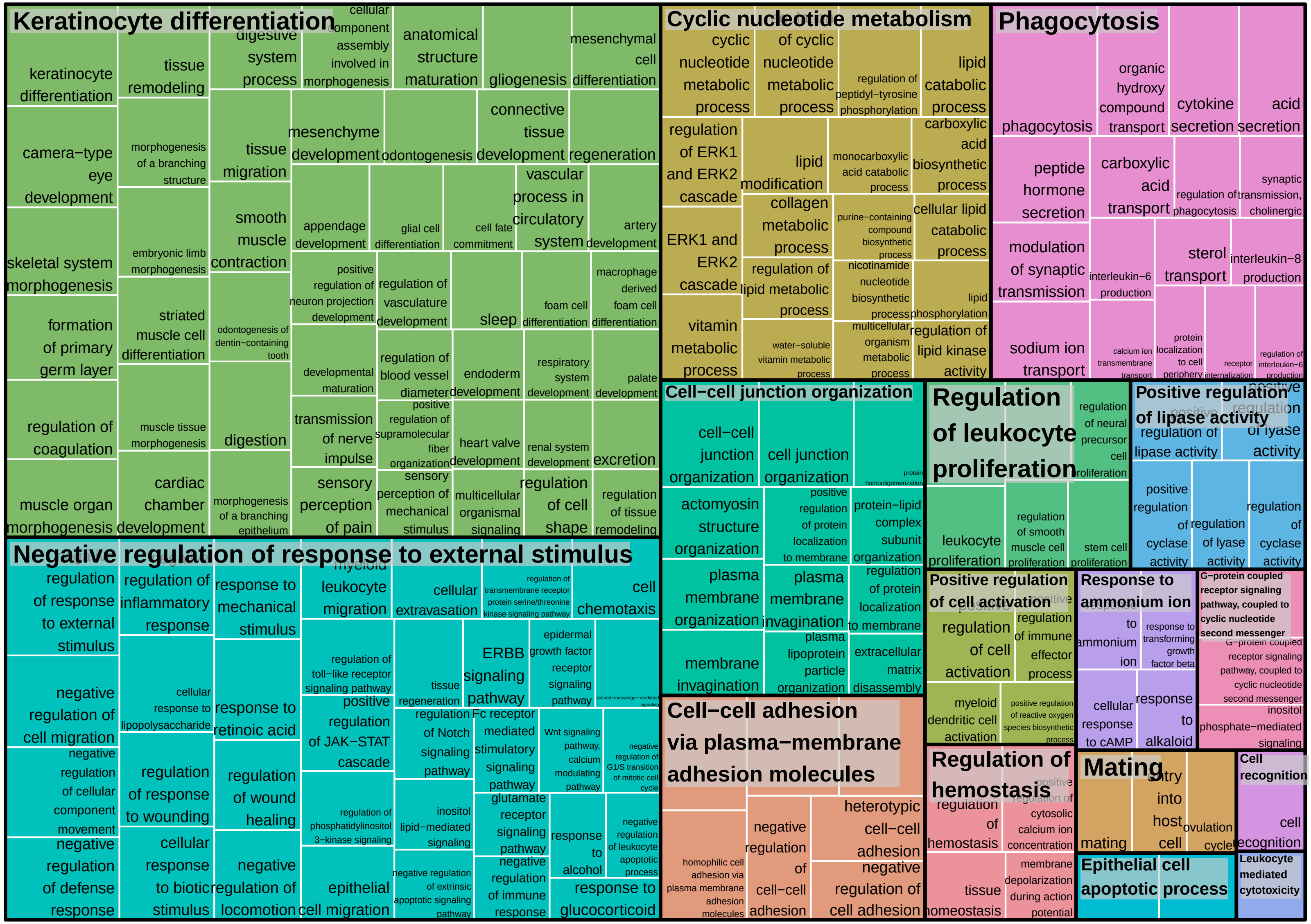

Supplementary Figure 10. REVIGO treemap of enriched gene ontologies from all cord tissue CpGs. Gene ontology (GO) enrichment was performed on missmethyl, with genes containing any of the 134,676 infant cord tissue CpGs analyzed, using all Infinium450K CpGs as the background. REVIGO was subsequently used to reclassify the biological process-related enriched GO terms (parent GO term containing under 300 genes, semantic similarity measure between each GO term < 0.7) resulting in 16 GO clusters from 174 unique GO terms. Each GO cluster is represented with a unique color with the cluster name on the top left corner enclosed by bold black outlines in the treemap while the individual GO terms are expounded on the bottom right corner enclosed by white outlines. The size of each treemap cell was automatically generated by REVIGO based on the statistical significances of the GO terms.

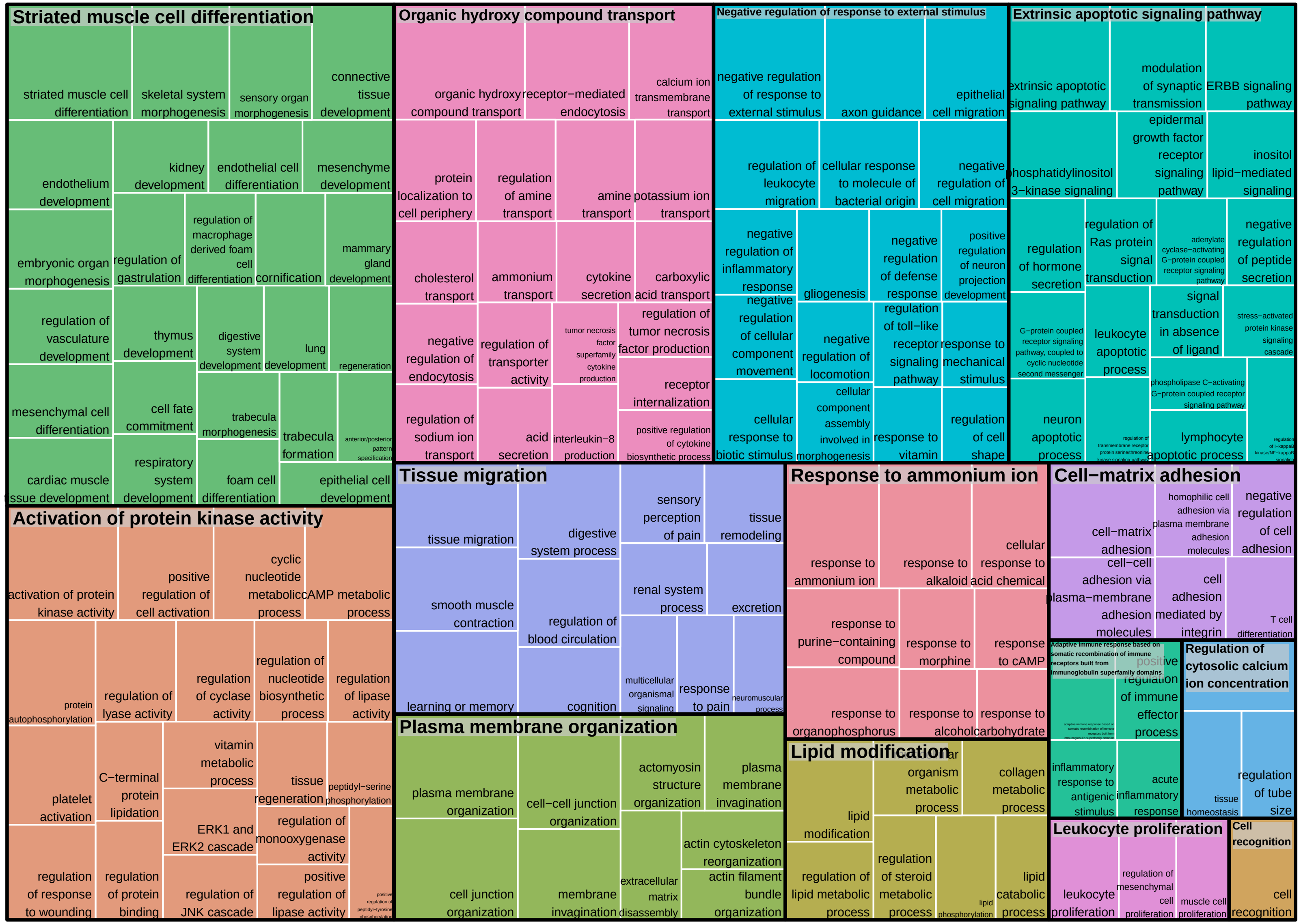

Supplementary Figure 11. REVIGO treemap of enriched gene ontologies from all cord blood CpGs. Gene ontology (GO) enrichment was performed on missmethyl, with genes containing any of the 85,624 infant cord blood CpGs analyzed, using all Infinium450K CpGs as the background. REVIGO was subsequently used to reclassify the biological process-related enriched GO terms (parent GO term containing under 300 genes, semantic similarity measure between each GO term < 0.7) resulting in 14 GO clusters from 160 unique GO terms. Each GO cluster is represented with a unique color with the cluster name on the top left corner enclosed by bold black outlines in the treemap while the individual GO terms are expounded on the bottom right corner enclosed by white outlines. The size of each treemap cell was automatically generated by REVIGO based on the statistical significances of the GO terms.

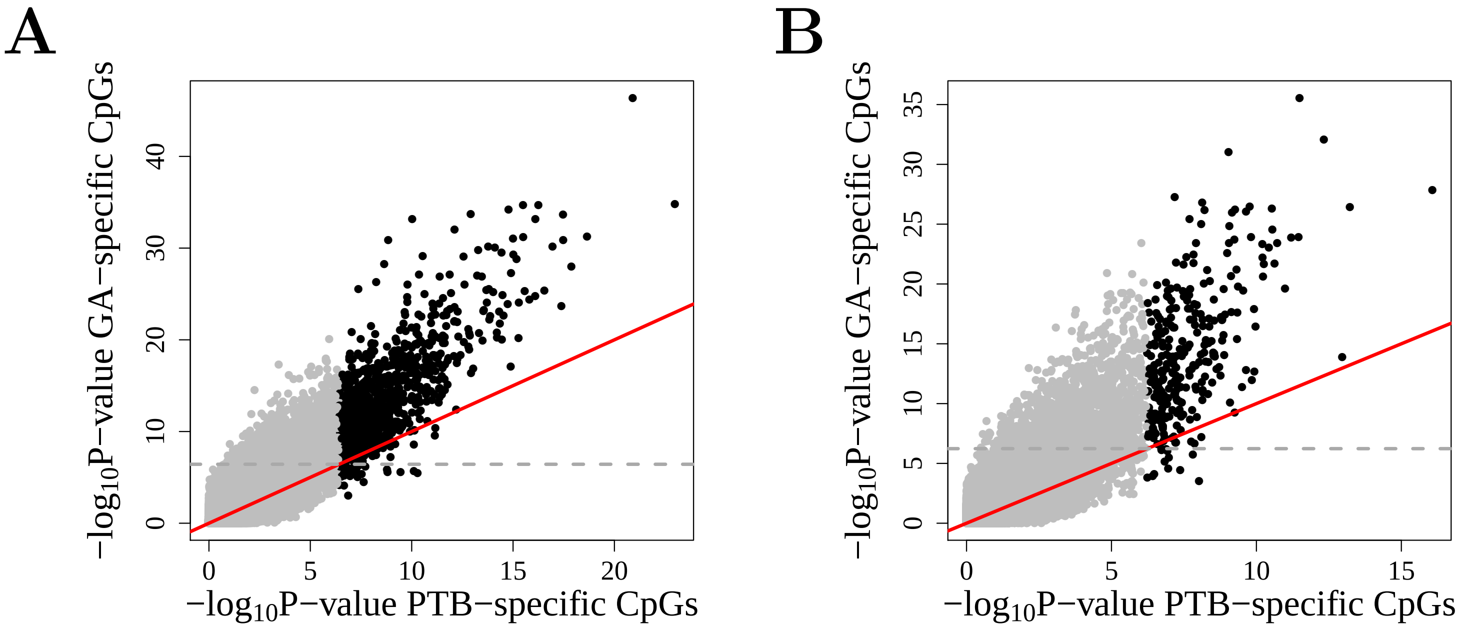

Supplementary Figure 12. Comparison of negative $\log_{10}$ p-values, from examining associations with DNA methylation (DNAm), using preterm birth (PTB) vs. using gestational age (GA). Scatterplots depict the negative logarithmic p-values for DNAm-GA associations using GA as a continuous variable (vertical axis) against negative logarithmic p-values for DNAm-PTB associations using PTB as a binary variable (horizontal axis) (**A**: cord tissue; **B**: cord blood).

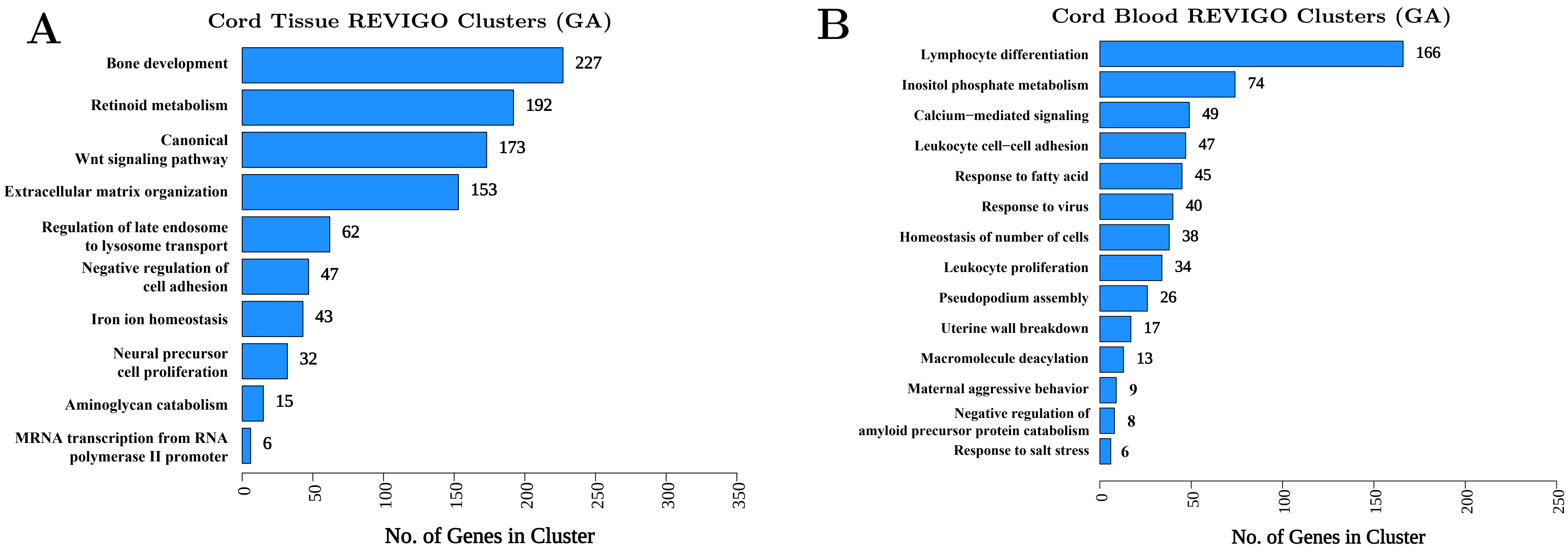

Supplementary Figure 13. REVIGO summarised Gene Ontology Clusters with respect to gestational age (GA)-associated CpGs in both cord tissue and cord blood. Gene ontology (GO) enrichment was performed on GA-associated CpGs in both cord tissue and cord blood, for each tissue separately, using missMethyl. REVIGO was then used to reclassify the biological process-related enriched GO terms (parent GO term containing under 300 genes, semantic similarity measure between each GO term < 0.7). Cord tissue CpGs had 11 GO clusters from 65 unique GO terms while cord blood CpGs had 14 GO clusters from 85 unique GO terms. GO clusters with 5 or more genes are represented by the bar graphs, with plots on the left and right corresponding to cord tissue (**A**) and cord blood (**B**) respectively. The vertical axis of the bar graphs represent the REVIGO cluster names while the horizontal axis represents the number of genes in the REVIGO cluster containing at least one significantly associated tissue-specific CpG.

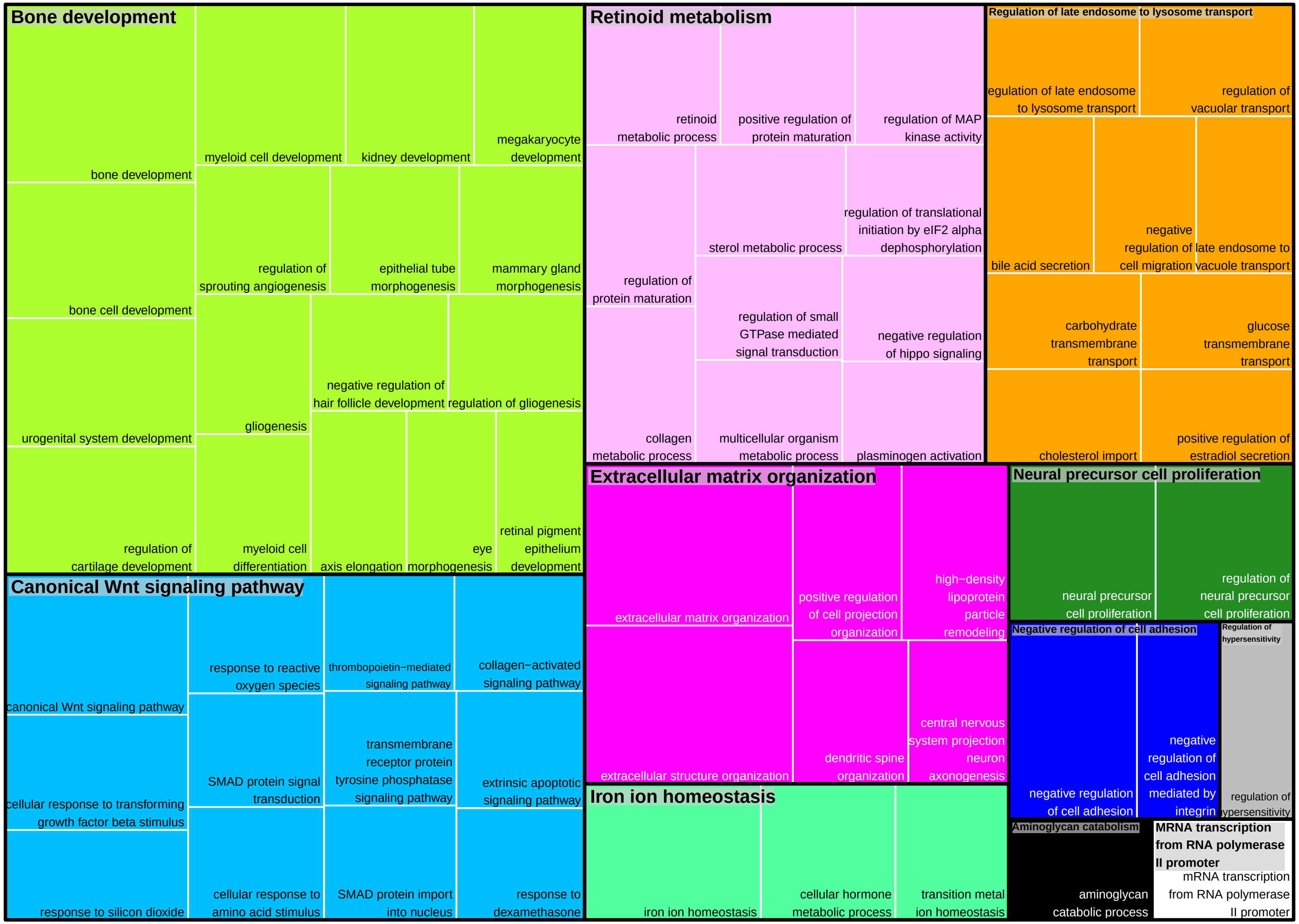

Supplementary Figure 14. REVIGO treemap of enriched gene ontologies from gestational age (GA)-associated cord tissue CpGs. Gene ontology (GO) enrichment was performed on genes containing any of the 4,075 significantly associated infant cord tissue CpGs, against genes lying in any of the 134,676 cord tissue CpGs analyzed as the background, with respect to GA using missMethyl. REVIGO was subsequently used to reclassify the biological process-related enriched GO terms (parent GO term containing under 300 genes, semantic similarity measure between each GO term < 0.7) resulting in 11 GO clusters from 65 unique GO terms. Each GO cluster is represented with a unique color with the cluster name on the top left corner enclosed by bold black outlines in the treemap while the individual GO terms are expounded on the bottom right corner enclosed by white outlines. The size of each treemap cell was automatically generated by REVIGO based on the statistical significances of the GO terms.

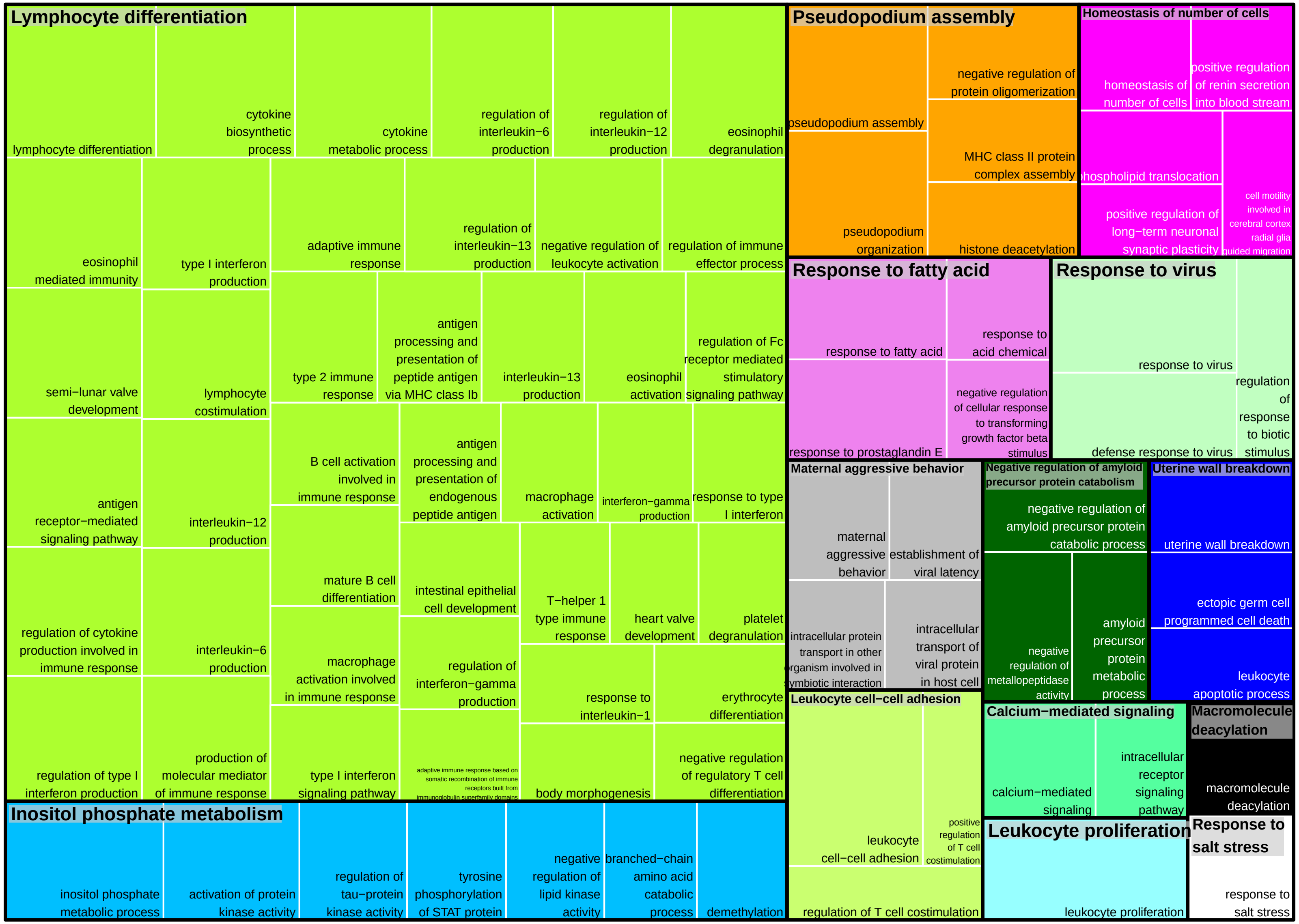

Supplementary Figure 15. REVIGO treemap of enriched gene ontologies from gestational age (GA)-associated cord blood CpGs. Gene ontology (GO) enrichment was performed on genes containing any of the 1,916 significantly associated infant cord blood CpGs, against genes lying in any of the 85,624 cord blood CpGs analyzed as the background, with respect to GA using missMethyl. REVIGO was subsequently used to reclassify the biological process-related enriched GO terms (parent GO term containing under 300 genes, semantic similarity measure between each GO term < 0.7) resulting in 14 GO clusters from 85 unique GO terms. Each GO cluster is represented with a unique color with the cluster name on the top left corner enclosed by bold black outlines in the treemap while the individual GO terms are expounded on the bottom right corner enclosed by white outlines. The size of each treemap cell was automatically generated by REVIGO based on the statistical significances of the GO terms.

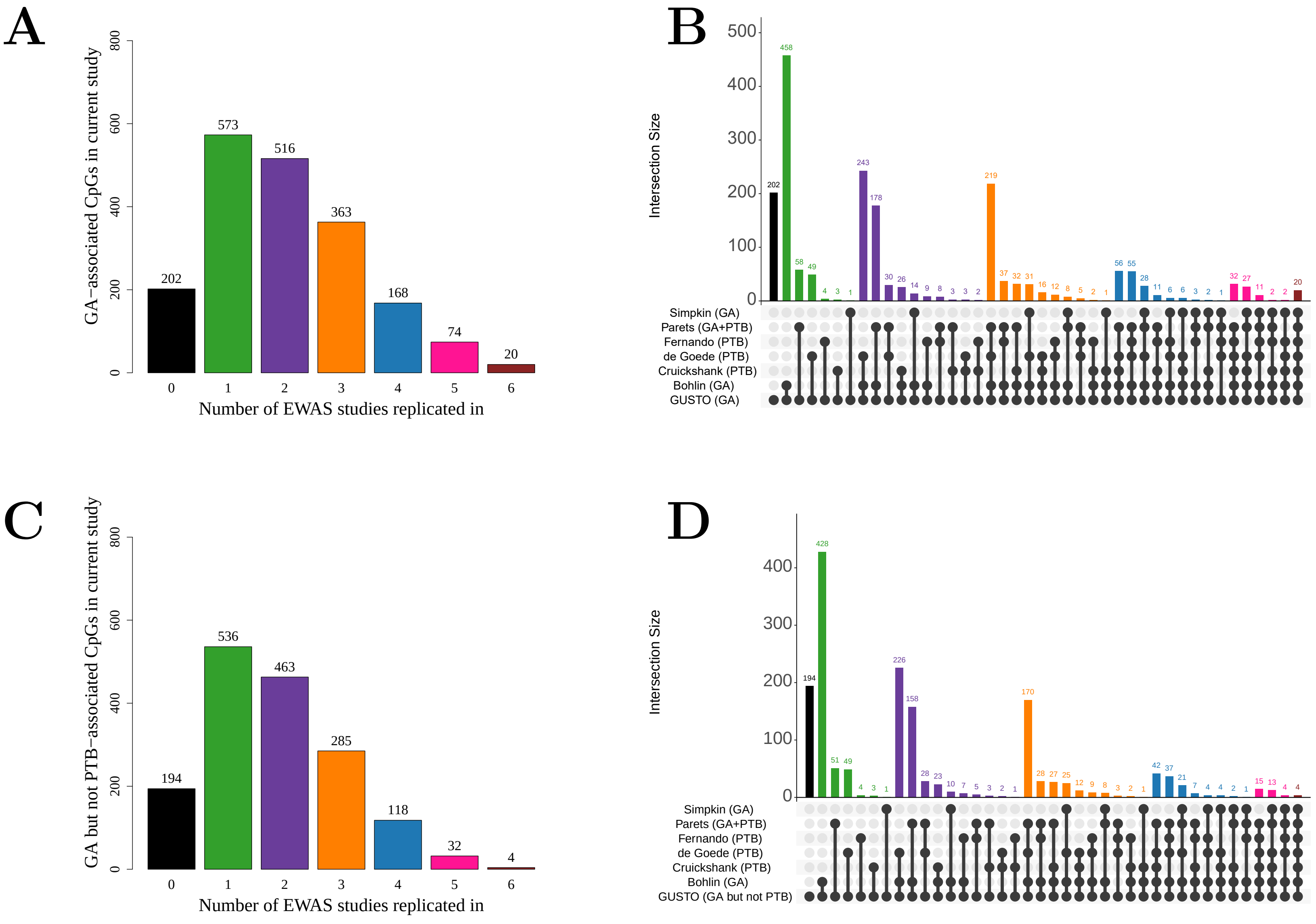

Supplementary Figure 16. Reproducibility of Cord Blood CpGs previously reported to associate with gestational age (GA) or preterm birth (PTB), with respect to GA-associated CpGs in the current study. In the current study, we found 1,916 CpGs to significantly associate with GA. **A** is a bar graph showing the reproducibility of the 1,916 GA-associated cord blood CpGs in the current study. The vertical axis gives the number of GA-associated cord blood CpGs in the current study while the horizontal axis gives the number of earlier GA/PTB epigenome-wide association studies (EWAS) our GA-associated cord blood CpGs are replicated in. Bar graph colors are representative of the number of earlier studies our GA-associated CpGs replicated in: black (0), green (1), purple (2), orange (3), blue (4), pink (5), and brown (6). **B** is an UpSet plot breaking down the replication of our GA-associated CpGs in the earlier studies. Each column represents the number of CpGs, for each unique intersection of the current study (GUSTO) with other studies, as indicated by the gray dot and connecting line. Intersection sets with no CpGs are not shown on the plot. As most (284/296) of our PTB-associated CpGs are also GA-associated, to distinguish GA but not PTB-associations, we plotted **C and D** with GA-but-not-PTB-associated cord blood CpGs in relation to the other studies as well.
