# Supplementary material for: Analysis of two birth tissues provides new insights into the epigenetic landscape of neonates born preterm
Source: Clin Epigenetics. 2019 Feb 11;11:26. doi: 10.1186/s13148-018-0599-4 (PMC6371604; doi:10.1186/s13148-018-0599-4)
Supplement: Supplementary file 2 — Supplementary tables. (ZIP 3966 kb) [file 13148_2018_599_MOESM2_ESM.zip › EWAS-PTB-Supplementary-Tables-v17.pdf]

Supplementary Tables

Version 17
Last updated: October 19, 2018

Supplementary Table 1. 1019 cord tissue characteristics of the GUSTO cohort studied in the analysis.

		Preterm (n=68) N (%)/Mean [SD]	Term (n=951) N (%)/Mean [SD]	Total (n=1019)
Ethnicity	Chinese	38 (56%)	553 (58%)	591 (58%)
	Indian	13 (19%)	165 (17%)	178 (17%)
	Malay	17 (25%)	233 (25%)	250 (25%)
Infant Sex	Male	35 (51%)	503 (53%)	538 (53%)
	Female	33 (49%)	448 (47%)	481 (47%)
Gestational age (days)		249 [12]	273 [7]	272 [10]

Supplementary Table 2. 332 cord blood characteristics of the GUSTO cohort studied in the analysis.

		Preterm (n=31) N (%)/Mean [SD]	Term (n=301) N (%)/Mean [SD]	Total (n=332)
Ethnicity	Chinese	19 (61%)	147 (49%)	166 (50%)
	Indian	5 (16%)	62 (21%)	67 (20%)
	Malay	7 (23%)	92 (31%)	99 (30%)
Infant Sex	Male	17 (55%)	148 (49%)	165 (50%)
	Female	14 (45%)	153 (51%)	167 (50%)
Gestational age (days)		249 [10]	273 [7]	271 [10]

Supplementary Table 3. 994 significant CpGs ($p < 3.7 \times 10^{-7}$) between preterm birth cases and term birth controls in 1019 cord tissue samples.

Table found in excel.

994 CpGs found Bonferroni significant (0.05/134,676 infant cord tissue CpGs analyzed = 3.7×10^{-7}) in the main analysis (cord tissue DNA methylation with respect to preterm birth status, adjusted for infant sex, ethnicity, cell-type proportions, bisulfite conversion batch, and DNA extraction batch) are listed in order of statistical significance alongside corresponding changes in DNA methylation Z-score (denoted as "Est"), 95% confidence intervals (95% CI) and p-values in sensitivity analysis 1 (cord tissue methylation with respect to preterm birth status, adjusted for infant sex, ethnicity, cell-type proportions, bisulfite conversion batch, DNA extraction batch, maternal age, maternal history of hypertension treatment, maternal exposure to cigarette smoke, mode of delivery, infant firstborn status, and chip position) and sensitivity analysis 2 (Surrogate Variable Analysis). CpGs are annotated with respect to known genomic features (gene names, position relative to CpG Islands and enhancers), analysis sample sizes (N), and GUSTO infant cord tissue DNA methylation Interquartile Ranges (IQR).

Supplementary Table 4. 296 significant CpGs ($p < 5.8 \times 10^{-7}$) between preterm birth cases and term birth controls in 332 cord blood samples.

Table found in excel.

296 CpGs found Bonferroni significant (0.05/85,624 infant cord blood CpGs analyzed = 5.8×10^{-7}) in the main analysis (cord blood DNA methylation with respect to preterm birth status, adjusted for infant sex, ethnicity, cell-type proportions, and bisulfite conversion batch) are listed in order of statistical significance alongside corresponding changes in DNA methylation Z-score (denoted as "Est"), 95% confidence intervals (95% CI) and p-values in sensitivity analysis 1 (cord blood methylation with respect to preterm birth status, adjusted for infant sex, ethnicity, cell-type proportions, bisulfite conversion batch, maternal age, maternal history of hypertension treatment, maternal exposure to cigarette smoke, mode of delivery, infant firstborn status, and chip position) and sensitivity analysis 2 (Surrogate Variable Analysis). CpGs are annotated with respect to known genomic features (gene names, position relative to CpG Islands and enhancers), analysis sample sizes (N), and GUSTO infant cord blood DNA methylation Interquartile Ranges (IQR).

Supplementary Table 5. Summary of previously published EWAS relating to preterm birth (PTB) or Gestational Age (GA).

S/N	First Author	Year	PMID	Study Type	Study Population	Sample Size (PT/T)	Sample Type	Platform	No. of Sig. PT CpGs	No. of Sig. GA CpGs
1	Parets	2013	23826308	PTB & GA	African Americans	50 (22/28)	fetal leukocyte DNA	INF450K	29	9637
2	Bohlin	2016	27717397	GA (ultrasound-estimated)	MoBa	1753	CB	INF450K	*n.a.*	5474
3	Simpkin	2015	25869828	GA	Aries, ALSPAC	914	CB	INF450K	*n.a.*	224
4	Fernando	2015	26419829	PTB	Dutch	22 (11/11, matched)	CB	INF450K	1347	*n.a.*
5	Cruickshank	2013	24134860	PTB (extreme)	Caucasian	24 (12/12, case control)	neonatal Guthrie cards (dried blood spots)	INF450K	1555	*n.a.*
6	de Goede	2017	28428831	PTB	Canadian	10 (5/5)	CB	INF450K	10,302 unique across all cell-types	*n.a.*

INF450K: Infinium Human Methylation 450K BeadChip. PMID: Pubmed Identification Number. PT: Number of Preterm subjects. T: Number of Term subjects.

Supplementary Table 6. Results of GUSTO cord blood preterm birth (PTB)-associated CpGs and previously published EWAS relating to preterm birth (PTB) or/and gestational age (GA).

Table found in excel.

23,066 unique CpGs with known assocations to PTB in the GUSTO study and associations to PTB and/or GA in previous studies mentioned in Supplementary Table 5. Associated effect sizes and statistical significances are only provided if the CpG passes statistical significance in their respective studies. "All (PTB+GA) Study Count" totals the previous citations for that particular CpG with respect to both PTB and GA. In "Bohlin (GA-u/s)", only CpGs associated with gestational age as determined by ultrasound are listed. In "de Goede (PT)", unique CpGs reported in any of the four cell-types investigated are listed. In "Parets", CpGs associated with GA and PTB are listed separately but are counted as one study in the "All (PTB+GA) Study Count" total.

Supplementary Table 7. Test for tissue-dependent effects for 448/994 cord tissue preterm birth (PTB)-associated CpGs ($p < 3.7 \times 10^{-7}$)

Table found in excel.

Results labeled under "Cord Tissue" are identical to those in Supplementary Table 3. Results labeled under "Cord Blood" give the corresponding DNAm-PTB association in cord blood, obtained by a linear regression analysis of cord blood DNAm against PTB, adjusted for covariates. Results labeled under "Interaction" give the interaction term from between PTB and tissue from fitting a general linear model with an unstructured covariance structure to a combined dataset with DNAm data from both tissues.

Supplementary Table 8. Test for tissue-dependent effects for 194/296 cord blood preterm birth (PTB)-associated CpGs ($p < 5.8 \times 10^{-7}$)

Table found in excel.

Results labeled under "Cord Blood" are identical to those in Supplementary Table 4. Results labeled under "Cord Tissue" give the corresponding DNAm-PTB association in cord tissue, obtained by a linear regression analysis of cord tissue DNAm against PTB, adjusted for covariates. Results labeled under "Interaction" give the interaction term from between PTB and tissue from fitting a general linear model with an unstructured covariance structure to a combined dataset with DNAm data from both tissues.

Supplementary Table 9. Enriched gene ontologies with respect to preterm birth-associated cord tissue CpGs.

S/N	GO ID	GO Term	N	DE	P.DE	REVIGO cluster
1	GO:0010718	positive regulation of epithelial to mesenchymal transition	39	6	7.5E-03	bone remodeling
2	GO:0010769	regulation of cell morphogenesis involved in differentiation	225	20	9.1E-03	bone remodeling
3	GO:0030282	bone mineralization	88	10	6.2E-03	bone remodeling
4	GO:0030325	adrenal gland development	18	5	2.1E-03	bone remodeling
5	GO:0031214	biomineral tissue development	115	13	2.1E-03	bone remodeling
6	GO:0033700	phospholipid efflux	12	3	9.1E-03	bone remodeling
7	GO:0036035	osteoclast development	16	5	2.0E-03	bone remodeling
8	GO:0046849	bone remodeling	68	10	8.5E-04	bone remodeling
9	GO:0060348	bone development	168	18	2.1E-03	bone remodeling
10	GO:1900040	regulation of interleukin-2 secretion	6	3	6.3E-03	bone remodeling
11	GO:0007178	transmembrane receptor protein serine/threonine kinase signaling pathway	284	24	1.5E-03	regulation of Wnt signaling pathway
12	GO:0007184	SMAD protein import into nucleus	22	5	2.0E-03	regulation of Wnt signaling pathway
13	GO:0007185	transmembrane receptor protein tyrosine phosphatase signaling pathway	7	3	1.0E-02	regulation of Wnt signaling pathway
14	GO:0007229	integrin-mediated signaling pathway	87	12	1.2E-03	regulation of Wnt signaling pathway
15	GO:0030111	regulation of Wnt signaling pathway	277	28	5.0E-05	regulation of Wnt signaling pathway
16	GO:0032488	Cdc42 protein signal transduction	8	3	3.1E-03	regulation of Wnt signaling pathway
17	GO:0032489	regulation of Cdc42 protein signal transduction	5	2	9.3E-03	regulation of Wnt signaling pathway
18	GO:0035582	sequestering of BMP in extracellular matrix	4	2	6.4E-03	regulation of Wnt signaling pathway
19	GO:0010903	negative regulation of very-low-density lipoprotein particle remodeling	3	2	2.0E-03	extracellular matrix organization
20	GO:0010989	negative regulation of low-density lipoprotein particle clearance	4	2	8.7E-03	extracellular matrix organization
21	GO:0030198	extracellular matrix organization	295	30	1.1E-04	extracellular matrix organization
22	GO:0043062	extracellular structure organization	296	30	1.2E-04	extracellular matrix organization
23	GO:0048251	elastic fiber assembly	6	3	3.2E-03	extracellular matrix organization
24	GO:0071800	podosome assembly	17	5	1.5E-03	extracellular matrix organization
25	GO:0002138	retinoic acid biosynthetic process	8	3	8.0E-03	regulation of aldosterone biosynthesis
26	GO:0006081	cellular aldehyde metabolic process	81	7	9.3E-03	regulation of aldosterone biosynthesis
27	GO:0006226	dUMP biosynthetic process	1	1	7.3E-03	regulation of aldosterone biosynthesis
28	GO:0008211	glucocorticoid metabolic process	23	4	9.5E-03	regulation of aldosterone biosynthesis
29	GO:0032347	regulation of aldosterone biosynthetic process	6	3	1.1E-03	regulation of aldosterone biosynthesis
30	GO:0001953	negative regulation of cell-matrix adhesion	26	5	7.4E-03	negative regulation of membrane protein ectodomain proteolysis
31	GO:0034446	substrate adhesion-dependent cell spreading	77	9	7.8E-03	negative regulation of membrane protein ectodomain proteolysis
32	GO:0045589	regulation of regulatory T cell differentiation	16	3	8.1E-03	negative regulation of membrane protein ectodomain proteolysis
33	GO:0051045	negative regulation of membrane protein ectodomain proteolysis	6	3	1.7E-03	negative regulation of membrane protein ectodomain proteolysis
34	GO:0071548	response to dexamethasone	37	6	4.2E-03	response to transforming growth factor beta
35	GO:0071559	response to transforming growth factor beta	198	19	8.6E-04	response to transforming growth factor beta
36	GO:1903844	regulation of cellular response to transforming growth factor beta stimulus	92	11	1.2E-03	response to transforming growth factor beta
37	GO:1990314	cellular response to insulin-like growth factor stimulus	6	2	9.7E-03	response to transforming growth factor beta
38	GO:0002931	response to ischemia	30	5	5.8E-03	response to ischemia
39	GO:0009399	nitrogen fixation	1	1	7.2E-03	nitrogen fixation
40	GO:0060013	righting reflex	8	3	9.5E-03	righting reflex
41	GO:0072672	neutrophil extravasation	7	4	2.1E-04	neutrophil extravasation

Gene ontology (GO) enrichment results from 994 Bonferroni significant preterm birth-associated cord tissue CpGs analyzed using missMethyl. GO terms relating to "Biological Processes" and containing under 300 genes in total (N), were further refined using REVIGO to remove GO terms with greater than 70% overlap in semantic similarity measure. DE: Number of genes associated with the Bonferroni significant cord tissue CpGs. P.DE: p-value significance of initial GO enrichment results from missMethyl. REVIGO cluster: GO grouping provided by REVIGO.

Supplementary Table 10. Enriched gene ontologies with respect to preterm birth-associated cord blood CpGs.

S/N	GO ID	GO Term	N	DE	P.DE	REVIGO cluster
1	GO:0001889	liver development	103	6	3.3E-03	regulation of T cell differentiation
2	GO:0002285	lymphocyte activation involved in immune response	114	6	8.6E-03	regulation of T cell differentiation
3	GO:0002286	T cell activation involved in immune response	65	6	5.7E-04	regulation of T cell differentiation
4	GO:0002367	cytokine production involved in immune response	66	5	3.3E-03	regulation of T cell differentiation
5	GO:0002440	production of molecular mediator of immune response	129	7	2.3E-03	regulation of T cell differentiation
6	GO:0002697	regulation of immune effector process	274	11	2.9E-03	regulation of T cell differentiation
7	GO:0002758	innate immune response-activating signal transduction	221	10	1.7E-03	regulation of T cell differentiation
8	GO:0002825	regulation of T-helper 1 type immune response	20	3	1.9E-03	regulation of T cell differentiation
9	GO:0032609	interferon-gamma production	83	6	3.5E-03	regulation of T cell differentiation
10	GO:0035710	CD4-positive, alpha-beta T cell activation	58	5	2.9E-03	regulation of T cell differentiation
11	GO:0042088	T-helper 1 type immune response	34	3	7.7E-03	regulation of T cell differentiation
12	GO:0045580	regulation of T cell differentiation	103	8	2.5E-04	regulation of T cell differentiation
13	GO:0060324	face development	40	4	9.0E-03	regulation of T cell differentiation
14	GO:0061008	hepaticobiliary system development	105	6	3.7E-03	regulation of T cell differentiation
15	GO:0007249	I-kappaB kinase/NF-kappaB signaling	221	9	4.3E-03	inositol lipid-mediated signaling
16	GO:0014066	regulation of phosphatidylinositol 3-kinase signaling	132	8	1.3E-03	inositol lipid-mediated signaling
17	GO:0043122	regulation of I-kappaB kinase/NF-kappaB signaling	201	8	9.2E-03	inositol lipid-mediated signaling
18	GO:0046580	negative regulation of Ras protein signal transduction	38	4	5.4E-03	inositol lipid-mediated signaling
19	GO:0048017	inositol lipid-mediated signaling	185	10	9.1E-04	inositol lipid-mediated signaling
20	GO:0051056	regulation of small GTPase mediated signal transduction	248	12	3.2E-03	inositol lipid-mediated signaling
21	GO:0051058	negative regulation of small GTPase mediated signal transduction	39	4	5.8E-03	inositol lipid-mediated signaling
22	GO:0009992	cellular water homeostasis	1	1	5.3E-03	regulation of RNA stability
23	GO:0043029	T cell homeostasis	29	3	3.2E-03	regulation of RNA stability
24	GO:0043487	regulation of RNA stability	108	6	3.5E-03	regulation of RNA stability
25	GO:0048872	homeostasis of number of cells	191	8	5.3E-03	regulation of RNA stability
26	GO:0051092	positive regulation of NF-kappaB transcription factor activity	106	6	9.2E-03	regulation of RNA stability
27	GO:2000653	regulation of genetic imprinting	1	1	6.2E-03	regulation of RNA stability
28	GO:0018095	protein polyglutamylation	8	2	8.8E-03	peptidyl-lysine deacetylation
29	GO:0031146	SCF-dependent proteasomal ubiquitin-dependent protein catabolic process	53	4	3.8E-03	peptidyl-lysine deacetylation
30	GO:0034983	peptidyl-lysine deacetylation	4	2	3.6E-03	peptidyl-lysine deacetylation
31	GO:0070933	histone H4 deacetylation	8	2	7.9E-03	peptidyl-lysine deacetylation
32	GO:0050766	positive regulation of phagocytosis	40	4	8.9E-03	positive regulation of extracellular matrix organization
33	GO:0071158	positive regulation of cell cycle arrest	68	5	2.6E-03	positive regulation of extracellular matrix organization
34	GO:1903055	positive regulation of extracellular matrix organization	16	3	2.4E-03	positive regulation of extracellular matrix organization
35	GO:0032527	protein exit from endoplasmic reticulum	29	3	6.3E-03	protein exit from endoplasmic reticulum
36	GO:0045210	FasL biosynthetic process	1	1	9.2E-03	FasL biosynthesis
37	GO:0045221	negative regulation of FasL biosynthetic process	1	1	9.2E-03	FasL biosynthesis
38	GO:0045332	phospholipid translocation	21	3	6.5E-03	protein exit from endoplasmic reticulum
39	GO:0046900	tetrahydrofolylpolyglutamate metabolic process	1	1	4.7E-03	tetrahydrofolylpolyglutamate biosynthesis
40	GO:0046901	tetrahydrofolylpolyglutamate biosynthetic process	1	1	4.7E-03	tetrahydrofolylpolyglutamate biosynthesis
41	GO:0070669	response to interleukin-2	7	2	3.6E-03	response to interleukin-2
42	GO:0071104	response to interleukin-9	1	1	9.2E-03	response to interleukin-2
43	GO:0010070	zygote asymmetric cell division	1	1	7.7E-03	zygote asymmetric cell division

Gene ontology (GO) enrichment results from 296 Bonferroni significant cord blood CpGs analyzed using missMethyl. GO terms relating to "Biological Processes" and containing under 300 genes in total (N), were further refined using REVIGO to remove GO terms with greater than 70% overlap in semantic similarity measure. DE: Number of genes associated with the Bonferroni significant cord tissue CpGs. P.DE: p-value significance of initial GO enrichment results from missMethyl. REVIGO cluster: GO grouping provided by REVIGO.

Supplementary Table 11. 4,075 significant CpGs ($p < 3.7 \times 10^{-7}$) gestational age (GA)-associated CpGs in 1019 cord tissue samples.

Table found in excel.

4,075 CpGs found Bonferroni significant (0.05/134,676 infant cord tissue CpGs analyzed = 3.7×10^{-7}) with respect to GA, adjusted for infant sex, ethnicity, cell-type proportions, bisulfite conversion batch, and DNA extraction batch) are listed in order of statistical significance alongside corresponding changes in DNA methylation Z-score per unit change in gestational age (denoted as "Est"), 95% confidence intervals (95% CI) and p-values.

Supplementary Table 12. 1,916 significant CpGs ($p < 5.8 \times 10^{-7}$) gestational age (GA)-associated CpGs in 332 cord blood samples.

Table found in excel.

1,916 CpGs found Bonferroni significant (0.05/85,624 infant cord blood CpGs analyzed = 5.8×10^{-7}) with respect to GA, adjusted for infant sex, ethnicity, cell-type proportions, and bisulfite conversion batch) are listed in order of statistical significance alongside corresponding changes in DNA methylation Z-score per unit change in gestational age (denoted as "Est"), 95% confidence intervals (95% CI) and p-values.

Supplementary Table 13. Enriched gene ontologies with respect to gestational age-associated cord tissue CpGs.

Table found in excel.

Gene ontology (GO) enrichment results from 4,075 Bonferroni significant gestational age-associated cord tissue CpGs analyzed using missMethyl. GO terms relating to "Biological Processes" and containing under 300 genes in total (N), were further refined using REVIGO to remove GO terms with greater than 70% overlap in semantic similarity measure. DE: Number of genes associated with the Bonferroni significant cord tissue CpGs. P.DE: p-value significance of initial GO enrichment results from missMethyl. REVIGO cluster: GO grouping provided by REVIGO.

Supplementary Table 14. Enriched gene ontologies with respect to gestational age-associated cord blood CpGs.

Table found in excel.

Gene ontology (GO) enrichment results from 1,916 Bonferroni significant gestational age-associated cord blood CpGs analyzed using missMethyl. GO terms relating to "Biological Processes" and containing under 300 genes in total (N), were further refined using REVIGO to remove GO terms with greater than 70% overlap in semantic similarity measure. DE: Number of genes associated with the Bonferroni significant cord tissue CpGs. P.DE: p-value significance of initial GO enrichment results from missMethyl. REVIGO cluster: GO grouping provided by REVIGO.

Supplementary Table 15. Results of GUSTO cord blood gestational age (GA)-associated CpGs and previously published EWAS relating to preterm birth (PTB) or/and gestational age (GA).

Table found in excel.

23,256 unique CpGs with known assocations to GA in the GUSTO study and associations to PTB and/or GA in previous studies mentioned in Supplementary Table 5. Associated effect sizes and statistical significances are only provided if the CpG passes statistical significance in their respective studies. "All (PTB+GA) Study Count" totals the previous citations for that particular CpG with respect to both PTB and GA. In "Bohlin (GA-u/s)", only CpGs associated with gestational age as determined by ultrasound are listed. In "de Goede (PT)", unique CpGs reported in any of the four cell-types investigated are listed. In "Parets", CpGs associated with GA and PTB are listed separately but are counted as one study in the "All (PTB+GA) Study Count" total.
